# Predicting in-hospital indicators from wearable-derived signals for cardiovascular and respiratory disease monitoring: An in silico study

**DOI:** 10.1371/journal.pdig.0001041

**Published:** 2025-10-14

**Authors:** Bianca Maria Laudenzi, Alberto Cucino, Sergio Lassola, Eleonora Balzani, Lucas Omar Müller

**Affiliations:** 1 Laboratory of Mathematics for Biology and Medicine, Department of Mathematics, University of Trento, Trento, Italy; 2 Department of Anesthesia and Intensive Care, Santa Chiara Hospital, APSS, Trento, Italy; 3 Centre for Medical Sciences-CISMed, University of Trento, Trento, Italy; Birjand University of Medical Sciences, IRAN, ISLAMIC REPUBLIC OF

## Abstract

Cardiovascular and respiratory diseases (CVRD) are the leading causes of death worldwide. The construction of health digital twins for patient monitoring is becoming a fundamental tool to reduce invasive procedures, lower healthcare costs, minimize patient hospitalization, design clinical trials and personalize therapies. The aim of this study is to investigate the feasibility of machine learning-based monitoring of healthy subjects and CVRD patients in an in silico context. A population of virtual subjects, both healthy and with CVRD, was created using a comprehensive zero-dimensional global closed-loop model. In particular, the most relevant model parameters were varied within physiologically and pathologically plausible ranges, using local sensitivity analysis to guide the parameter selection. Then, we trained Gaussian process regression (GPR) models, informed by wearable-acquired data (e.g., heart rate, peripheral pressures and oxygen saturation), to predict variables normally acquired with invasive or operator-dependent methods (e.g., central venous pressure, stroke volume, cardiac output, left ventricular ejection fraction, arterial partial pressure of O_2_, arterial partial pressure of CO_2_). We also evaluated GPR models performance under simulated wearable signal acquisition errors via an error propagation analysis. Presented results demonstrate the feasibility of predicting in-hospital variables from wearable-derived indices using GPR models under the controlled conditions and assumptions of the adopted modeling approach.

## Author summary

Cardiovascular and respiratory diseases are major global health concerns. Effective remote monitoring is essential for early detection of complications and improved patient care, especially for people with chronic conditions. Wearable devices provide a non-invasive way to track health indicators, but they do not directly measure certain key physiological parameters that doctors typically assess in hospitals. In this study, we explore how machine learning approaches can help bridge this gap. By using virtually-generated data, we trained Gaussian process regression models to estimate critical cardiovascular and respiratory indexes, such as cardiac output and oxygen levels. In particular, we created a virtual population of simulated patients and used their data to train and test our model. Our findings suggest that this approach can be a valuable tool for remote monitoring, providing healthcare professionals with accurate insights, without the need for invasive procedures and enabling earlier detection of complications. However, further testing with real patient data is necessary to fully assess its clinical potential.

## Introduction

According to the World Health Organization [1], noncommunicable diseases account for more than 80 % of premature deaths. Cardiovascular and cardio-respiratory diseases (CVRD) account for the majority of non-communicable deaths (43 % and 10 % respectively), followed by cancer (21 %), and diabetes (4 %). Given the significant impact of CVRD on global mortality, effective monitoring of these diseases is essential for early detection and timely intervention. For this reason, the healthcare industry is witnessing an increase in the prevalence of wearable technologies since their impact on CVRD management has become undeniable [2–5]. The use of wearable technology in addressing CVRD offers significant clinical benefits such as decreasing healthcare costs, reducing the need for patients’ hospitalization, minimizing invasive procedures, designing clinical trials and personalized therapies, providing accurate long-term monitoring of cardiac indices during daily activities and sleep [3]. However, only a limited number of bio-signals can be estimated easily and accurately via commercial wearable devices, such as heart rate (HR), blood pressures (BPs), and arterial O_2_ saturation (S_a,O_2__) [4,5], all derived using a photoplethysmogram (PPG), which is an optical non-invasive technique that uses a light-emitting diode to illuminate a capillary bed to monitor pulsatile changes in light absorption [6]. For many devices, their acquisition accuracy meets the FDA standards. Table 1 reports FDA-approved commercial wearable devices as well as their accuracy percentage for all wearable derived signals, i.e. HR, BPs, and S_a,O_2__. However, the clinical parameters required to monitor CVRD effectively extend beyond these signals. These include central venous pressure (CVP), stroke volume (SV), cardiac output (CO), left ventricular ejection fraction (EF), arterial partial pressure of O_2_ (P_a,O_2__), arterial partial pressure of CO_2_ (P_a,CO_2__).

**Table 1:** Summary of variables for CVRD monitoring and their acquisition methods.

Variable		Gold-standard	W-device [type] (A)	W-technology
**W-derived**	HR	ECG	• OMRON [7] [Watch] (± 5 %) • MOCAcuff [8] [Band] (± 5 %)	PPG
	BPs	Intra-arterial catheterization	• OMRON [7] [Watch] (± 3 mmHg*) • MOCAcuff [8] [Band] (± 3 mmHg*)	PPG
	S_a,O_2__	Blood gas analysis	• Oxitone [9] [Wrist-sensor] (± 2 %) • iHealth [10] [Fingertip] (± 2 %)	PPG
**Variable**		**Gold-standard**	**Possible technologies for W-devices**	
**In-hospital**	CVP	Jugular vein catheterization	PPG [11–14]	
	SV	Fick, thermodilution	Arterial waveform [15–20]	
	CO	Fick, thermodilution	Arterial waveform [15–20]	
	EF	Cardiac catheterization	No available technology	
	P_a,O_2__	Blood gas analysis	NN using HR and S_a,O_2__ [21]	
	P_a,CO_2__	Blood gas analysis	No available technology	

Abbreviations: W: wearable; A: accuracy; ECG: electrocardiography; PPG: photoplethysmography; NN: neural networks; HR: heart rate; BPs: arterial blood pressures; S_a,O_2__: arterial O_2_ saturation; CVP: central venous pressure; SV: left ventricle stroke volume; CO: left ventricle cardiac output; EF: left ventricle ejection fraction; P_a,O_2__: arterial partial pressure of O_2_; P_a,CO_2__: arterial partial pressure of CO_2_. Note: we include only wearable devices that are FDA-approved and have an available accuracy. * = validated against the mercury sphygmomanometer, considered the primary reference in blood pressure measurement.

CVP is used in clinical practice to assess volume status and cardiac preload, and its monitoring is crucial to understand and follow the hemodynamic status of patients with cardio-respiratory diseases. The gold standard technique for CVP measurement is invasive, requiring catheterization of the jugular vein [22]. However, well-accepted non-invasive options are ultrasound-guided techniques [23–25]. Some preliminary studies used PPG techniques to estimate CVP [11–14]. While promising, these findings were derived from relatively small sample sizes in specific surgical environments (perioperative or anesthesiology settings), indicating a need for broader validation across diverse clinical settings. Additionally, the need for precise positioning and multiple measurement points prevents existing methods from being automated and easily applicable, particularly in non-specialized home-based care, making this a significant limitation.

CO, SV, and EF are key indicators for evaluating cardiac circulatory failure and monitoring overall cardiac function [26,27]. Methods for monitoring SV and CO are categorized into gold-standard invasive techniques (e.g., methods based on the Fick principle, thermodilution, pulse-indicated continuous CO method [28]) and non-invasive approaches (e.g., partial CO_2_ repeated respiration [29], nuclear magnetic resonance method [30], pulse contour analysis method [31] or studies on relationship between blood flow and skin temperature [32]). However, most of the latter are only suitable for single or short-term measurements and are not appropriate for 24-hour continuous and remote monitoring. More recent model-based methods [15–20] refine SV and CO estimation using features of the arterial peripheral waveform or more complex mathematical models based on waveform shape. Such approaches are promising for their implementation into wearable devices, but further development is needed to make these methods widely applicable in the wearable industry. EF can be measured invasively during cardiac catheterization by contrast left ventriculography [27], or non-invasively via imaging modalities such as echocardiography, TAC, or MRI [33–35]. Overall, the current non-invasive methods for EF remain operator-dependent and are unsuitable for use in a home setting.

Monitoring P_a,O_2__ and P_a,CO_2__ is essential to follow patients with acute respiratory failure such as hypercapnia and hypoxia, respectively [36]. Arterial blood gas analysis is the gold standard technique for such a purpose, but it is invasive, intermittent, and potentially painful [37]. Some studies estimate P_a,O_2__ non-invasively and continuously using pulse rate and S_a,O_2__, by means of nonlinear mixed effects regression analysis [38] or, more recently, neural networks [21]. However, both these studies collect data only from pediatric patients, limiting them to hospitalized conditions and small age ranges. Regarding P_a,CO_2__, a non-invasive and remote technique uses transcutaneous sensors based on the principle of the Severinghaus electrode [39]. However, such monitors are not only expensive but also bulky and continuously drifting, requiring frequent recalibration by trained medical staff. Therefore, the current emphasis is on the development of wearable and easily deployable devices for remote patient monitoring outside clinical settings [37,40].

From this literature review we have identified a set of clinically relevant variables for CVRD monitoring that are predominantly measured in hospital settings, each obtained through distinct acquisition techniques (i.e., CVP, CO, SV, EF, P_a,O_2__, and P_a,CO_2__). Throughout this text, we will refer to these as in-hospital variables. To the best of our knowledge, no comprehensive and general solution exists that predicts these in-hospital variables for remote monitoring. The challenge lies in predicting these clinical indicators remotely using a common methodology across all variables, leveraging wearable biosignals, such as HR, systolic and diastolic BPs, and S_a,O_2__, without requiring substantial new hardware or changing the acquisition technique of the wearable device. The development and validation of such methodology typically require a large set of measurements from real human subjects with sufficient variety. Such data collection can be a very arduous and expensive task. In this work, we performed a preliminary assessment of the approach, which can be conducted without the need for extensive data collection, using virtual populations (VPs) generated through mathematical models, as demonstrated in similar studies [17,41,42]. In this context, each virtual patient is defined as a snapshot, on the order of seconds, of clinically relevant physiological variables. The snapshot is intended to represent a physiologically plausible state at a specific point in time, analogous to a single clinical measurement taken from an individual.

## Materials and methods

**Fig 1:**
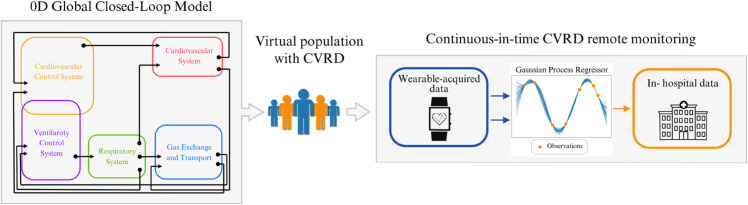
Graphical abstract. A virtual database is created using a comprehensive 0D global closed-loop cardio-respiratory model. Such database of bio-signals allowed us to explore Gaussian process regressor’s potential to predict in-hospital variables using wearable-acquired bio-signals.

The methodology followed in this work is summarized in Fig 1. In particular, a virtual population was generated using a zero-dimensional (0D) global closed-loop cardio-respiratory model. This database of bio-signals enabled us to investigate the potential of Gaussian process regression (GPR) models in predicting in-hospital variables from wearable-acquired signals, in an in silico setting.

### Global closed-loop cardio-respiratory model

**Fig 2:**
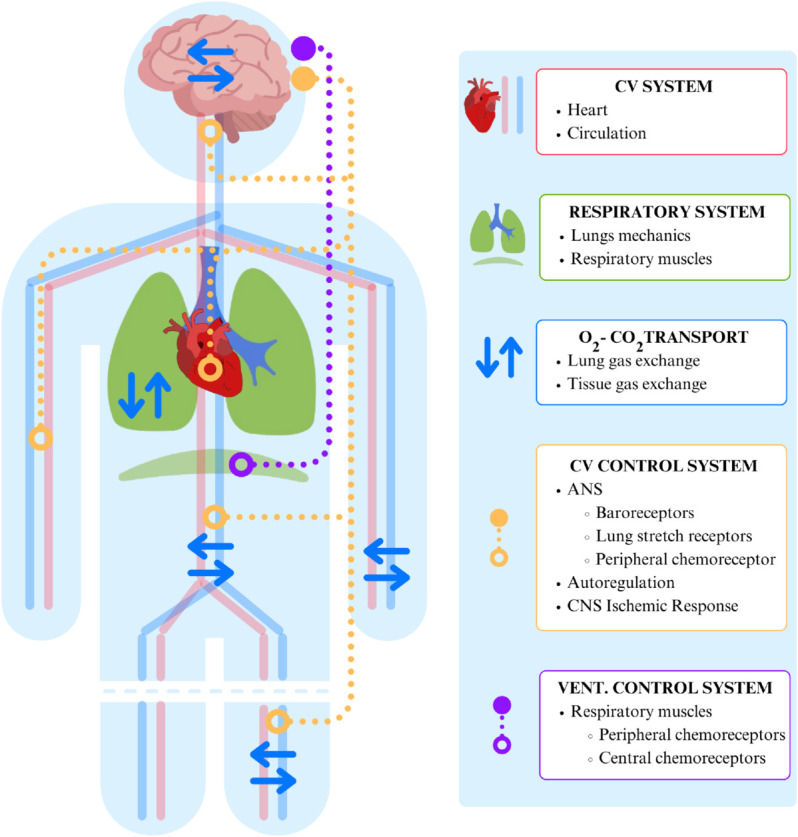
Schematic representation of the cardio-respiratory model. The figure shows the interaction between the cardiovascular and respiratory systems, highlighting gas exchange processes and control mechanisms. Abbreviations: CV: cardiovascular, ANS: autonomic nervous system, CNS: central nervous system, VENT.: ventilatory.

The bio-signals of interest were simulated using a 0D global closed-loop mathematical model comprising major elements characterizing cardiovascular function, such as blood flow in heart chambers, systemic and pulmonary circulation, respiration and gas transport and metabolisms, as well as main short-term regulatory mechanisms. The selected model is an extension of model [43] and consists of a non-linear system of differential-algebraic equations. The model equations can be found in S1 Appendix. Fig 2 shows a schematic representation of the cardio-respiratory model.

Numerical integration of differential equations was performed using the fourth-order Runge-Kutta method with a time step of 5 ⋅ 10^−5^ s, using an in-home code implemented in C++. For all simulations, model output signals were saved between 1940 and 2000 s, with a sampling period of 0.01 s. Clinically relevant variables were then extracted from this interval. Extracted model output variables included the wearable-derivable signals such as HR, central systolic/diastolic BPs (CSBP/CDBP), S_a,O_2__, the in-hospital ones, i.e. CVP, SV, CO, EF, P_a,O_2__, and P_a,CO_2__ and other signals such as central pulse pressure (CPP), and mean arterial pressure (MAP). A precise description of the computation of the indexes is provided in S2 Appendix. Briefly, CDBP, MAP, and CSBP were identified respectively as the minimum, the mean, and the maximum of the arterial pressure waveform $P_{sa}$ over each cardiac cycle. CPP was computed as the difference between CSBP and CDBP over each cardiac cycle. CVP, CO, S_a,O_2__, P_a,O_2__ and P_a,CO_2__ were computed respectively as the mean over each cardiac cycle of the thoracic vein pressure curve $P_{tv}$, the flow curve through the aortic valve $q_{AV}$, the arterial O_2_ saturation waveform $S_{a,O_{2}}$, the arterial partial pressure curve of O_2_ $P_{a,O_{2}}$, and the arterial partial pressure curve of CO_2_ $P_{a,CO_{2}}$. SV was computed as the product of CO and heart period for each cardiac cycle. Left ventricle end-diastolic and systolic volumes (LVEDV and LVESV) were identified respectively as the maximum and the minimum of the left ventricle volume curve $V_{LV}$ over each cardiac cycle. EF was computed as the difference between LVEDV and LVESV over LVEDV, for each cardiac cycle. All indexes were averaged over the interval 1940–2000 s.

### Virtual database generation

A virtual database of clinically relevant variables for CVRD monitoring was generated by varying the most sensitive model input parameters. These parameters were identified through a local sensitivity analysis. Then we assigned uniform distributions to the selected parameters, using physiological and pathological ranges. The parameter space was then sampled effectively, and the cardio-respiratory model was run at each sampled point.

A post-processing filter was applied to ensure the physiological or pathological plausibility of the model output variables and that simulations reached a stationary state. The time scale of our model is on the order of seconds and, as such, is designed to capture fast physiological dynamics, i.e. cardiac, respiratory, and short-term regulatory cycles. Therefore, we consider the system to be in a stationary state when the average values of clinically relevant variables, computed over time intervals longer than the characteristic periods of the included physiological dynamics, achieve constant values.

CVRD-affected bio-signals were classified within the VP, ensuring the generation of both healthy and CVRD-affected patients.

#### Model parameters selection.

For the identification of the most influential parameters, we explored the local sensitivity of clinically relevant model output variables to all 286 model input parameters. A simple definition of the local sensitivity $S_{P}^{M}$ of model output M to changes in a certain parameter P is based on derivatives, i.e.

$$S_{P}^{M}=\frac{\hat{P}}{\hat{M}}\frac{\partial M(P)}{\partial P},$$ (1)

where $\hat{P}$ and $\hat{M}$ denote the baseline values for the considered parameter and model output [44]. In particular, we approximated the partial derivative in Eq 1 using centered finite differences, and we chose to conduct the analysis by considering a 10 % variation below and above the baseline parameter value $\hat{P}$.

To ensure a comprehensive representation of the cardio-respiratory system, we selected parameters influencing various physiological domains based on their significance in the local sensitivity analysis (see Table 6). The selected parameters are: the total blood volume ($V_{tot}$) and the venous unstressed volume ($V_{u,ven}$) since they ranked as the most relevant parameters for multiple clinically relevant outputs; the basal cardiac cycle (*T*_0_) and the gain in vagal stimulus for heart period ($G_{T_{v}}$), since they affect HR; the carotid baroreflex activation level ($P_{n}$) since it is the most influential parameter representing a feedback mechanism; the basal maximum active elastance for the left ventricle ($E_{max,LV0}$) as it is the most influential parameter representing the heart contractility; the inspired O_2_ fraction ($F_{{iO}_{2}}$), the hemoglobin content (*hgb*), the maximum CO_2_ saturation ($C_{sat,CO2}$), and the empirical parameter for CO_2_ dissociation ($h_{{CO}_{2}}$), since they are the fundamental parameters describing gas exchange and transport. This selection ensured coverage of critical aspects of cardiovascular and respiratory physiology while focusing on parameters to which clinically relevant outputs are highly sensitive.

#### Generation of the virtual database.

Parameters selected on the basis of local sensitivity analysis were considered as random variables with uniform distribution. The lower and upper bounds were based on ranges reported in the literature, as summarized in Table 2. Specifically, the assignment of some ranges is based on the following assumptions. We assumed that the percentage variation of $V_{u,ven}$ matched that of $V_{tot}$. Given that the carotid baroreflex control system would typically adjust its set-point to maintain homeostasis in response to sustained changes in MAP, the baroreflex activation level $P_{n}$ is assumed to vary proportionally with MAP. Furthermore, since the heart period increases linearly with efferent vagal frequency [45], it seemed physiologically reasonable to modify both the basal heart rate (*T*_0_) and the gain in vagal stimulus for the heart period ($G_{T_{v}}$) simultaneously.

**Table 2:** Physiological ranges of input parameters for the generation of VP 1.

Parameter	Notation	Distribution	Units	Reference
Total blood volume	$V_{tot}$	𝒰(4373.0,6164.4)	mL	[46]
Venous unstressed volume	$V_{u,ven}$	𝒰(2574.9,3629.6)	mL	[46]*
Basal cardiac cycle	*T*_0_***	𝒰(0.41,0.59)	s	[47]
Gain in vagal stimulus for heart period	$G_{T_{v}}$***	𝒰(0.07,0.11)	$s\cdot \nu^{-1}$	[45]
Baroreflex activation level	$P_{n}$	𝒰(81.9,102.1)	mmHg	[47]**
Basal maximum active elastance of left ventricle	$E_{max,LV0}$	𝒰(1.67,3.11)	mmHg ⋅ mL^−1^	[50]
Inspired fraction of O_2_	$F_{{iO}_{2}}$	𝒰(14.7,27.3)	%	[43]
Blood hemoglobin content	*hgb*	𝒰(0.15,0.19)	$g\cdot {mL}^{-1}$	[51]
Maximum concentration of hemoglobin-bound CO_2_	$C_{{sat,CO}_{2}}$	𝒰(1.7,2.2)	${mL}_{O_{2}}\cdot {mL_{blood}}^{-1}$	[52]
Empirical parameters for CO_2_ dissociation curve	$h_{{CO}_{2}}$	𝒰(1.7,1.9)	-	[52]

Note: ν = spikes/s;   = we assumed that $V_{u,ven}$ has the same percentage variation as $V_{tot}$; ** = we assumed that the baroreflex activation level $P_{n}$ has the same variation of MAP; *** = we modify both *T*_0_ and $G_{T_{v}}$ simultaneously [45].

We sampled the 9^th^-dimensional parameter space employing a quasi-Monte Carlo sampling strategy based on Sobol’ sequences with scrambling, implemented via the *qmc.Sobol* function from the *SciPy* library. This method generates low-discrepancy samples in the multidimensional parameter space, ensuring uniform coverage of the domain [53]. The cardio-respiratory model was executed for every point in the sampled parameter space. Convergence of the number of sampling points used to generate the VP was verified running the model with 128, 1024, 4096, 8192 sampling points and comparing means, standard deviations (SDs), minimums and maximums of all clinically relevant variables. The errors for convergence were computed with respect to the highest number of sampling points as

$$\frac{\left|e_{n}-e_{8192}\right|}{\left|e_{8192}\right|}\cdot 100,\;where\;n=\{128,1024,4096\},$$ (2)

and *e* represents either the mean, the SD, the minimum, or the maximum of a specific variable.

#### Filter criteria and CVRD classification.

Following [54], we applied a post-processing filter to ensure that each numerical simulation reached a stationary state and remained within the physiological ranges representative of healthy or CVRD-affected individuals. In particular, we excluded simulations meeting either one of the following criteria: (1) any clinically relevant model output variable showed a difference greater than 1 % between the data collected in the intervals 1940–2000 s and 2940–3000 s of the simulation, or (2) any variable fell outside its specified physiological or pathological range. The admissible ranges for each variable are provided in Table 3.

**Table 3:** Filter criteria for model output variables.

Variable	Min	Max	Unit	Reference
HR	45	94	$beats\cdot {min}^{-1}$	[47]
CSBP	84	174	mmHg	[47]
CDBP	59	110	mmHg	[47]
CPP	13	86	mmHg	[47]
MAP	74	132	mmHg	[47]
CVP	0	10	mmHg	[47]
SV	44	128	mL	[55]
CO	42	150	$mL\cdot s^{-1}$	[55]
EF	45	74	%	[56]
S_a,O_2__	80	100	%	[57]
P_a,O_2__	70	110	mmHg	[57]
P_a,CO_2__	30	50	mmHg	[57]

Abbreviations: HR: heart rate; CSBP/CDBP: central systolic/diastolic blood pressure; CPP: central pulse pressure; MAP: mean arterial pressure; CVP: central venous pressure; SV: left ventricle stroke volume; CO: left ventricle cardiac output; EF: left ventricle ejection fraction; S_a,O_2__: arterial O_2_ saturation; P_a,O_2__: arterial partial pressure of O_2_; P_a,CO_2__: arterial partial pressure of CO_2_.

Furthermore, since we wanted to create a virtual database representative of both healthy and CVRD-affected patients, we classified simulations that passed the filtering stage into healthy or CVRD-affected bio-signals, such as bradycardia, hypertension, hypo and hyperventilation and reduced cardiac function, according to the cutoff values reported in Table 4. Notably, a single simulation could be classified under multiple pathological conditions.

**Table 4:** CVRD-classification for model output variables.

Variable	Cutoff	Unit	CVRD	Reference
HR	< 60	$beats\cdot {min}^{-1}$	Bradycardia	[58]
CSBP	> 130	mmHg	Hypertension	[59]
CDBP	> 80	mmHg	Hypertension	[59]
MAP	> 100	mmHg	Hypertension	[59]
EF	< 50	%	Reduced cardiac function	[60]
P_a,CO_2__	< 35	mmHg	Hyperventilation	[59]
P_a,CO_2__	> 45	mmHg	Hypoventilation	[59]

Abbreviations: HR: heart rate; CSBP/CDBP: central systolic/diastolic blood pressure; EF: left ventricle ejection fraction; P_a,CO_2__: arterial partial pressure of CO_2_.

### Prediction of in-hospital variables

#### Gaussian process regression.

We used GPR for predicting in-hospital data by leveraging bio-signals that can be acquired through wearable devices, in accordance with the literature review summarized in Table 1. GPR was selected due to its suitability in biomedical and personalized healthcare applications [61–63]. Several comparative studies have shown that GPR models outperforms or matches neural networks, support vectors machines and decision tree models in terms of predictive accuracy, especially in low-data, high-uncertainty scenarios [64–66]. Moreover, GPR offers improved interpretability, requires minimal hyperparameter tuning, and allows the incorporation of prior knowledge via kernel design [67–69]. In addition, GPR provides not only accurate predictions but also uncertainty estimates, which are crucial in clinical contexts. Briefly, a GPR model is a statistical model that describes how a scalar simulator output f(𝐱) varies as a function of *D* input features $𝐱=(x_{1},...,x_{D})$. The simulator outputs, f(·), are modeled as being jointly Gaussian with prior mean

m(𝐱)=𝔼[f(𝐱)], (3)

and covariance

$$k(𝐱,{𝐱}^{\prime })=\mathbb{C}[f(𝐱),f({𝐱}^{\prime })],$$ (4)

where $k(𝐱,{𝐱}^{\prime })$ is a positive-definite kernel [67]. In this case, the GPR model training is done in Python with *Scikit Learn* [70]. For each GRP model, the kernel is the product of a Constant kernel (*sklearn.gaussian_process.kernels.ConstantKernel*) and a Radial Basis Function kernel (*sklearn.gaussian_process.kernels.RBF*), in which the hyperparameters are tuned using the function *sklearn.model_selection.RandomizedSearchCV* with 10-fold cross-validation with random shuffling of the data and maximum error scoring strategy (*sklearn.metrics.max_error*).

After selecting the simulations that have not failed the filter criteria, the virtual dataset VP 1 is divided into 80 % training (1431 samples) and 20 % test (358 samples). We train independently six GPR models on the normalized training set $({𝐗}_{TR},{𝐲}_{TR})=\{({𝐱}^{i},y^{i}){\}}_{i=1}^{1431}$, where ${𝐗}_{TR}$ is the input features matrix of wearable-derived signals, with

$${𝐱}^{i}=\left[{HR}^{i},{CSBP}^{i},{CDBP}^{i},{S_{a,O_{2}}}^{i}\right],$$ (5)

while ${𝐲}_{TR}$ represents the vector of one of the six target variables that we aim to predict independently, i.e.

$$y^{i}\in \{{CVP}^{i},\;{SV}^{i},\;{CO}^{i},\;{EF}^{i},\;{P_{a,CO_{2}}}^{i},\;{P_{a,O_{2}}}^{i}\}.$$ (6)

The performance of the GPR models were then evaluated on the test set $({𝐗}_{GT},{𝐲}_{GT})=\{({𝐱}^{j},y^{j}){\}}_{j=1}^{358}$, exploiting the standard scoring metrics: the coefficient of determination *R*^2^, the percentage maximum relative error, i.e.

maxRE=max(RE), (7)

and the percentage mean relative error, i.e.

MRE=mean(RE), (8)

where **RE** is the percentage relative error.

To assess the effect of the training set size on the performance of the GPR models, learning curves were computed for training sizes of 100, 200, 300, 500, and 1000 data points, sampled from the VP 1 train set. For this purpose, we used the *sklearn.model_selection.learning_curve* function with 10-fold cross-validation with random shuffling of the data and maximum error scoring strategy (*sklearn.metrics.max_error*).

#### Error propagation analysis.

The input features provided to the GPR models are signals acquired through wearable devices, which inherently include acquisition errors. In order to account for this in our work, we assumed acquisitions errors of ± 5 % for HR [7,8] and ± 2 % for S_a,O_2__ [9,10]. For BP signals we needed to account not only for the errors introduced by the wearable devices but also for the errors resulting from estimating central BP from peripheral BP measurements using transfer functions. Indeed, while the cardio-respiratory model accurately represents central BPs, which are the ones used in the input vector features, i.e. CSBP and CDBP, it does not provide precise peripheral BP values. The mean bias of transfer functions, compared to gold-standard measurements, ranges between 1-5 mmHg [71–73], whereas the error introduced by the wearable-device acquisition process is ± 3 mmHg [7,8]. As a result, we assumed an overall error of ± 3 mmHg for CSBP and CDBP. Given these acquisition errors, we conducted an error propagation analysis for each trained GPR model to assess the impact of these errors on the model’s performance. The error propagation analysis represents an average-case approximation that provides a baseline understanding of model sensitivity to input uncertainty, while recognizing that wearable device errors in real-world applications may exhibit individual-specific and non-stationary characteristics.

In particular, assuming that the errors are distributed uniformly, for each test data point *j*, with j=1,...,358, we sampled *N*-times the multidimensional uniform distribution of input features with their acquisition errors, using a quasi-Monte Carlo sampling strategy based on Sobol’ sequences with scrambling, implemented via the *qmc.Sobol* function from the *SciPy* library. Thus, we obtained the sample set of vectors $\chi^{j}$, i.e.

$$\chi^{j}=\{\chi_{k}^{j}{\}}_{k=1}^{N}\sim 𝒰({𝐱}^{j}-\epsilon^{j},\;{𝐱}^{j}+\epsilon^{j}),$$ (9)

where

$${𝐱}^{j}=\left[{HR}^{j},{CSBP}^{j},{CDBP}^{j}{S_{a,O_{2}}}^{j}\right]$$ (10)

is the input features vector for the test data point *j* and

$$\epsilon^{j}=[5\text{ \% of }{HR}^{j},\;3\;\text{mmHg},\;3\;\text{mmHg},\;2\text{ \% of }{S_{a,O_{2}}}^{j}]$$ (11)

is the corresponding input reference acquisition errors ranges.

Finally, for each test data point *j*, we evaluated the six trained GPR models on the sample set $\chi^{j}$, obtaining the corresponding sample set of predictions ${𝒴}^{j}=\{{𝒴}_{k}^{j}{\}}_{k=1}^{N}$, with

$$𝒴\in \{CVP,\;SV,\;CO,\;EF,\;P_{a,CO_{2}},\;P_{a,O_{2}}\}.$$ (12)

The performance of the GPR models predictions with perturbed input features was evaluated on the test set $({𝐗}_{GT},{𝐲}_{GT})=\{({𝐱}^{j},y^{j}){\}}_{j=1}^{358}$ using the following metrics. The percentage maximum relative error

$$maxRE_{PI}=max({RE}_{PI}),$$ (13)

the percentage mean relative error

$$MRE_{PI}=mean({RE}_{PI}),$$ (14)

and the coefficient of variation

$$CV=\frac{\sigma }{𝐄}\cdot 100,$$ (15)

where ${RE}_{PI}$ is the vector of percentage relative errors with perturbed input features defined as

$${RE}_{PI}=\frac{\left|𝐄-{𝐲}_{GT}\right|}{\left|{𝐲}_{GT}\right|}\cdot 100,$$ (16)

with $𝐄=[\begin{array}{c} 𝔼[{𝒴}^{j}] \end{array}{]}_{j=1}^{358}$ being the vector of estimated expected values of $\{{𝒴}_{k}^{j}{\}}_{k=1}^{N}$ and $\sigma =[\begin{array}{c} \sigma [{𝒴}^{j}] \end{array}{]}_{j=1}^{358}$ representing the vector of estimated SDs of $\{{𝒴}_{k}^{j}{\}}_{k=1}^{N}$.

Different sampling sizes N={50,100,500,1000} of $\{\chi_{k}^{j}{\}}_{k=1}^{N}$ were used to ensure convergence of the methodology comparing means and SDs of the vector of percentage relative errors ${RE}_{PI}$.

#### Validation against new virtual population.

To ensure that the predictive capabilities of our model were not overly reliant on the parameters choice that we made for the generation of VP 1, we tested the GPR models, which were trained on the VP 1 dataset, on a new dataset. This new population was called VP 2 and was obtained by varying different combinations of 0D model parameters. In this case, we selected the model input parameters analyzing again the results of the local sensitivity analysis (see Table 6) but using a different criteria: we chose all the parameters that ranked in the first 3 ranking positions, i.e. the total blood volume ($V_{tot}$), the blood hemoglobin content (*hgb*), the maximum concentration of hemoglobin-bound CO_2_ ($C_{{sat,CO}_{2}}$), the venous unstressed volume ($V_{u,ven}$), the ventricles elastances ($E_{max,LV0}$, $k_{E,LV}$, $k_{E,RV}$), the inspired fraction of O_2_ ($F_{{iO}_{2}}$), the empirical parameters for the O_2_/CO_2_ dissociation curve ($h_{O_{2}}$, $h_{{CO}_{2}}$, $k_{{CO}_{2}}$) and the basal cardiac cycle (*T*_0_). Following the same procedure as before, we assigned uniform distributions to the selected model parameters, based on the physiological and pathological ranges summarized in Table 5, then we sampled the parameter space employing the *qmc.Sobol* function from the *SciPy* library. The cardio-respiratory model was run at each sampled point. Note that the ventricles elastances ($E_{max,LV0}$, $k_{E,LV}$ and $k_{E,RV}$) were modified simultaneously, resulting in a 10^th^-dimensional parameter space.

**Table 5:** Physiological ranges of input parameters for the generation of VP 2.

Parameter	Notation	Distribution	Unit	Reference
Total blood volume	$V_{tot}$	𝒰(4373.0,6164.4)	mL	[46]
Venous unstressed volume	$V_{u,ven}$	𝒰(2574.9,3629.6)	mL	[46]
Basal cardiac cycle	*T*_0_	𝒰(0.41,0.59)	s	[47]
Inspired fraction of O_2_	$F_{{iO}_{2}}$	𝒰(14.7,27.3)	%	[43]
Blood hemoglobin content	*hgb*	𝒰(0.15,0.19)	$g\cdot {mL}^{-1}$	[51]
Basal maximum active elastance of left ventricle	$E_{max,LV0}$**	𝒰(1.79,2.99)	mmHg ⋅ mL^−1^	[50]
Passive elastance of left ventricle	$k_{E,LV}$**	𝒰(0.01,0.02)	mL^−1^	[74]
Passive elastance of right ventricle	$k_{E,RV}$**	𝒰(0.008,0.014)	mL^−1^	[74]
Maximum concentration of hemoglobin-bound CO_2_	$C_{{sat,CO}_{2}}$	𝒰(1.7,2.2)	$mL_{O_{2}}\cdot {mL_{blood}}^{-1}$	[52]
Empirical parameter for O_2_ dissociation curve	$h_{O_{2}}$	𝒰(0.38,0.39)	-	[52]
Empirical parameter (1) for CO_2_ dissociation curve	$h_{{CO}_{2}}$	𝒰(1.7,1.9)	-	[52]
Empirical parameter (2) for CO_2_ dissociation curve	$k_{{CO}_{2}}$	𝒰(110,279)	-	[52]

Note: ν = spikes/s;   = we assumed that $V_{u,ven}$ has the same percentage variation as $V_{tot}$; ** = $E_{max,LV0}$, $k_{E,LV}$ and $k_{E,RV}$ are modified simultaneously.

The post-processing filter criteria defined in Table 3 as well as the CVRD classification scheme reported in Table 4 were applied. After selecting the simulations that did not fail the filter criteria, the six GPR models were tested on VP 2. Following the same procedure described in section Error propagation analysis, we tested the GPR models with perturbed input data of VP 2.

## Results

First, we examine output variables from the 0D cardio-respiratory model, focusing on validation, feature extraction, and local sensitivity analysis. Then, we address the results of the VPs, focusing on convergence, global sensitivity analysis, variables distributions and post-processing filters, as well as a comparison between VP 1 and VP 2. Lastly, we explore performance of the GPR models predictions for the in-hospital variables.

### Cardio-respiratory model.

The 0D model was validated in the baseline case by comparing the model-predicted cardiac and hemodynamic indices with reference values reported in the literature. For a detailed comparison between the model predictions and the literature data, please refer to S1 Appendix.

**Fig 3:**
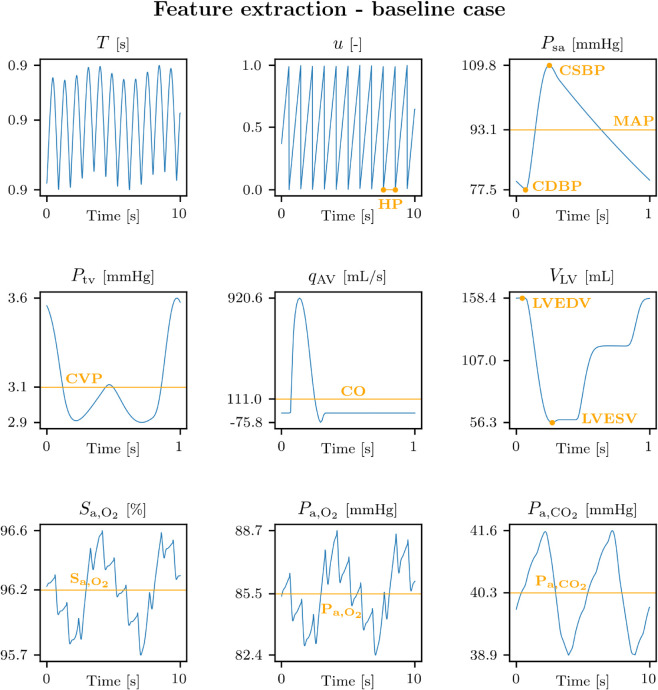
Cardio-respiratory model features extraction. Signals over time (blue curves) and extracted features (orange indexes). The y-axes indicate the minimum, the mean, and the maximum values for each signal. Abbreviations: *T*: heart cycle duration; *u*: fraction of the cardiac cycle; $P_{sa}$: systemic arterial pressure; $P_{tv}$: thoracic vein pressure; $q_{AV}$: flow though the aortic valve; $V_{LV}$: left ventricle volume; $P_{a,O_{2}}$: arterial O_2_ saturation; $P_{a,O_{2}}$ arterial partial pressure curve of O_2_; $P_{a,CO_{2}}$: arterial partial pressure curve of CO_2_; HP: heart period; CSBP/CDBP/MAP: central systolic/diastolic/mean blood pressure; CVP: central venous pressure; CO: left ventricle cardiac output; LVESV/LVEDV: left ventricle end systolic/diastolic volume; S_a,O_2__: arterial O_2_ saturation; P_a,O_2__: arterial partial pressure of O_2_; P_a,CO_2__: arterial partial pressure of CO_2_.

Fig 3 shows model output variables over time (blue lines) and extracted features (orange indexes) for the 0D cardio-respiratory model in the baseline case. The first plot displays the heart cycle duration, *T*, which exhibits the expected variations due to the interaction of sympathetic and vagal activity over the shorter timescale and respiratory modulation over the longer timescale. The range values close to 0.9 s are consistent with a normal resting heart rate [47]. The dimensionless variable *u* represents the fraction of the cardiac cycle and allows for the identification of each heart period, with *u* = 0 assumed at the beginning of systole. The systemic arterial pressure, $P_{sa}$, displays a physiological pulsatile pattern, with extracted features for CSBP, CDBP, and MAP all within normal ranges [47]. Similarly, the thoracic vein pressure, $P_{tv}$, reflects the expected CVP range with small oscillations around a stable baseline value [75]. The flow through the aortic valve, $q_{AV}$, shows a typical systolic ejection phase, with a slightly elevated peak flow, along with a brief period of backward flow at the beginning of diastole. This behavior is due to including non-ideal valves in the model, representing a modification compared to the previous approach [43] that assumed ideal valves. The left ventricular volume, $V_{LV}$, follows the expected pattern of filling and emptying of the ventricle, with extracted features for LVEDV and LVESV volumes yielding a normal stroke volume [76]. The arterial oxygen saturation, $S_{a,O_{2}}$, shows small oscillations around a stable baseline value, while the partial pressures of oxygen $P_{a,O_{2}}$ and carbon dioxide $P_{a,CO_{2}}$ oscillate within physiological ranges due to respiratory cycles [57].

Table 6 shows the local sensitivity analysis results of all 286 model inputs for some selected model outputs, i.e. the wearable-acquirable signals HR, CSBP, CDBP, and S_a,O_2__, and the in-hospital ones, CVP, SV, CO, EF, P_a,O_2__, and P_a,CO_2__. Only the parameters ranked within the first five ranking positions, together with their sensitivity index $S_{P}^{M}$, are reported. The total blood volume $V_{tot}$ and the venous unstressed volume $V_{u,ven}$ appear to be the most influential parameters across all signals. In addition, CVP, SV, CO and EF show high sensitivity to ventricular elastance parameters ($k_{E,LV}$, $k_{E,RV}$, and $E_{max,LV0}$), highlighting the critical role of cardiac mechanics. HR is mainly influenced by the basal hear rate (*T*_0_) and the gain in vagal stimulus ($G_{Tv}$), while CSBP and CDBP are sensitive to the maximum concentration of hemoglobin-bound CO_2_ ($C_{{sat,CO}_{2}}$) and the empirical parameter for the CO_2_ dissociation curve ($h_{{CO}_{2}}$). Respiratory outputs (S_a,O_2__, P_a,O_2__, P_a,CO_2__) are primarily affected by the maximum concentration of hemoglobin-bound CO_2_ ($C_{{sat,CO}_{2}}$), the hemoglobin concentration (*hgb*) and the inspired oxygen fraction ($F_{{iO}_{2}}$). These findings inform the selection of key parameters for generating VP 1 and VP 2.

**Table 6:** Local sensitivity analysis for the cardio-respiratory model.

Variable	Rank 1	Rank 2	Rank 3	Rank 4	Rank 5
HR	$V_{tot}$ (-1.2)	$V_{u,ven}$ (0.6)	*T*_0_ (-0.4)	$G_{T_{v}}$ (-0.4)	$P_{n}$ (0.3)
CSBP	$V_{tot}$ (1.9)	$V_{u,ven}$ (-1.1)	$C_{{sat,CO}_{2}}$ (-0.5)	$h_{{CO}_{2}}$ (-0.5)	$P_{n}$ (0.4)
CDBP	$V_{tot}$ (1.6)	$V_{u,ven}$ (-0.9)	$C_{{sat,CO}_{2}}$ (-0.8)	$h_{{CO}_{2}}$ (-0.7)	$P_{n}$ (0.6)
S_a,O_2__	*hgb* (-0.5)	$F_{{iO}_{2}}$ (0.1)	$h_{O_{2}}$ (-0.1)	$V_{tot}$ (-0.1)	$k_{O_{2}}$ (-0.1)
CVP	$V_{tot}$ (8.0)	$V_{u,ven}$ (-4.8)	$k_{E,RV}$ (1.0)	$V_{u,art}$ (-0.9)	$P_{l}$ (0.9)
SV	$V_{tot}$ (2.9)	$V_{u,ven}$ (-1.7)	$k_{E,LV}$ (-0.4)	$V_{u,art}$ (-0.3)	$k_{E,RV}$ (-0.3)
CO	$V_{tot}$ (2.0)	$V_{u,ven}$ (-1.1)	$k_{E,LV}$ (-0.2)	*hgb* (-0.2)	$k_{E,RV}$ (-0.2)
EF	$V_{tot}$ (0.4)	$E_{max,LV0}$ (0.3)	$V_{u,ven}$ (-0.2)	$C_{{sat,CO}_{2}}$ (0.2)	$h_{CO_{2}}$ (0.2)
P_a,O_2__	$C_{{sat,CO}_{2}}$ (-1.2)	$F_{{iO}_{2}}$ (1.0)	$h_{{CO}_{2}}$ (-1.0)	$V_{tot}$ (-0.8)	$P_{atm}$ (0.7)
P_a,CO_2__	$C_{{sat,CO}_{2}}$ (-1.6)	$h_{{CO}_{2}}$ (-1.5)	$k_{{CO}_{2}}$ (0.9)	$\alpha_{{CO}_{2}}$ (0.7)	$\beta_{{CO}_{2}}$ (-0.6)

Parameters are ranked from the highest absolute sensitivity score, annotated in round brackets, down to the fifth ranking position. Variables abbreviations: HR: heart rate; CSBP/CDBP: central systolic/diastolic blood pressure; S_a,O_2__: arterial O_2_ saturation; SV: left ventricle stroke volume; CO: left ventricle cardiac output; EF: left ventricle ejection fraction; P_a,O_2__: arterial partial pressure of O_2_; P_a,CO_2__: arterial partial pressure of CO_2_. Parameters abbreviations: $V_{tot}$: total blood volume; $V_{u,ven}$ venous unstressed blood volume; *T*_0_: basal cardiac cycle; $G_{T_{v}}$: gain in vagal stimulus for heart period; $P_{n}$: baroreflex activation level; $C_{{sat,CO}_{2}}$: maximum concentration of hemoglobin-bound CO_2_; $h_{{CO}_{2}}$, $h_{O_{2}}$, $k_{O_{2}}$, $k_{{CO}_{2}}$, $\alpha_{{CO}_{2}}$, $\beta_{{CO}_{2}}$: empirical parameters for O_2_/CO_2_ dissociation curves; *hgb*: blood hemoglobin content; $F_{{iO}_{2}}$: inspired fraction of O_2_; $k_{E,LV}$, $k_{E,RV}$, $E_{max,LV0}$: parameters for left and right ventricles elastances; $V_{u,art}$: arterial unstressed blood volume; $P_{l}$: pleural pressure; $P_{atm}$: atmospheric pressure.

### Virtual populations.

In this section, we report the results of VP 1, similar results for VP 2 are omitted here for brevity but can be found in S3 Appendix.

**Fig 4:**
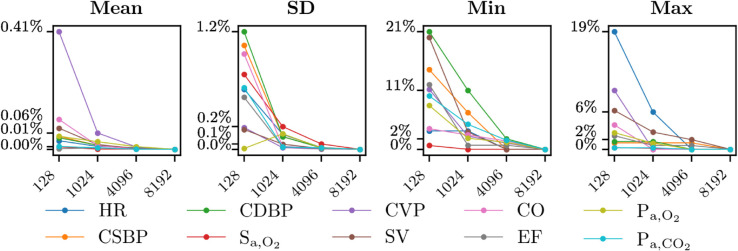
Convergence of the number of sampling points. Percentage errors of means, standard deviations (SDs), minimums and maximums for all variables of interest of VP 1 (y-axes), for different sampling points sizes (x-axes). Abbreviations: HR: heart rate; CSBP/CDBP: central systolic/diastolic blood pressure; S_a,O_2__: arterial O_2_ saturation; SV: left ventricle stroke volume; CO: left ventricle cardiac output; EF: left ventricle ejection fraction; P_a,O_2__: arterial partial pressure of O_2_; P_a,CO_2__: arterial partial pressure of CO_2_.

Fig 4 illustrates the convergence of the variables of interest for different sampling point numbers (128, 1024, 4096, and 8192), specifically reporting percentage errors of means, SDs, minimums, and maximums for all clinically relevant variables of VP 1. For the means and SDs, strong convergence was observed across all variables, with maximum error variations of 0.01 % and 0.1 %, respectively, at 4096 sampling points. Convergence was slightly more challenging to achieve for minimums and maximums, which both showed maximum errors of 2% across all variables at 4096 sampling points. Overall, we conclude that 4096 sampling points are sufficient to ensure convergence, as the errors for all statistical indicators remain acceptably low.

**Fig 5:**
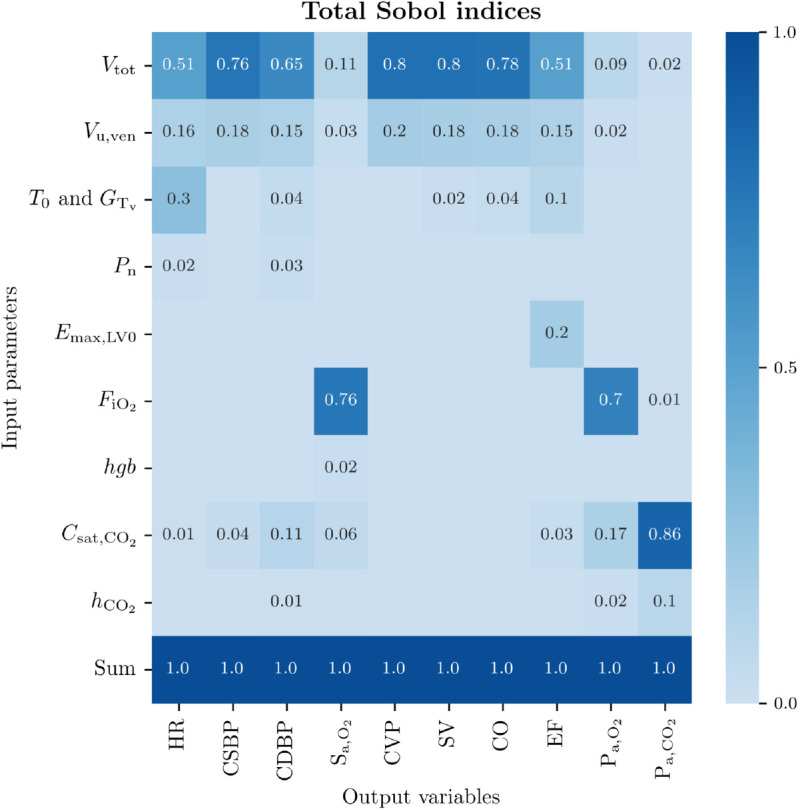
Global sensitivity analysis. Total effects of input parameters (y-axis) on model outputs (x-axis), normalized between 0 and 1, for VP 1. Parameters abbreviations: $V_{tot}$: total blood volume; $V_{u,ven}$ venous unstressed volume; *T*_0_: basal cardiac cycle; $G_{T_{v}}$: gain in vagal stimulus for heart period; $P_{n}$: baroreflex activation level; $E_{max,LV0}$: basal maximum elastance of the left ventricle; $F_{{iO}_{2}}$: inspired fraction of O_2_; *hgb*: blood hemoglobin content; $C_{{sat,CO}_{2}}$: maximum concentration of hemoglobin-bound CO_2_; sh: pulmonary shunt; $h_{{CO}_{2}}$ : empirical parameters for CO_2_ dissociation curve. Variables abbreviations: HR: heart rate; CSBP/CDBP: central systolic/diastolic blood pressure; S_a,O_2__: arterial O_2_ saturation; SV: left ventricle stroke volume; CO: left ventricle cardiac output; EF: left ventricle ejection fraction; P_a,O_2__: arterial partial pressure of O_2_; P_a,CO_2__: arterial partial pressure of CO_2_.

A global sensitivity analysis was performed on the 9 parameters varied for generating VP 1, using the *analyze.enhanced_hdmr.analyze* function form the *SALib* library [77]. Fig 5 shows the heat map of the total Sobol indices for the selected 9 model input parameters over all clinically relevant output variables of VP 1 generated with 4096 sampling points. Darker colors refer to higher interaction between inputs and outputs. The indexes for each parameter were normalized so that they sum up to 1, representing 100 % of the output variance. Indexes lower than 0.01 were not annotated in the heat map. The variance-based global sensitivity analysis identified the total blood volume, $V_{tot}$, and the venous unstressed volume, $V_{u,ven}$, as the most significant parameters for a subset of selected signals of the cardiovascular system, i.e. HR, CSBP, CDBP, CVP, SV, CO, EF. Moreover, the basal heart period, *T*_0_, together with the gain in vagal stimulus for heart period, $G_{T_{v}}$, also had a significant influence on HR and EF. In addition, EF is also influenced by the basal maximum elastance of the left ventricle $E_{max,LV0}$. On the other hand, the inspired fraction of O_2_, $F_{{iO}_{2}}$, and the CO_2_ saturation concentration, $C_{{sat,CO}_{2}}$, were the most significant parameters for the cardio-respiratory bio-signals, i.e. S_a,O_2__, P_a,O_2__ and P_a,CO_2__. These results confirm the relevance of the parameters identified in the local sensitivity analysis.

**Fig 6:**
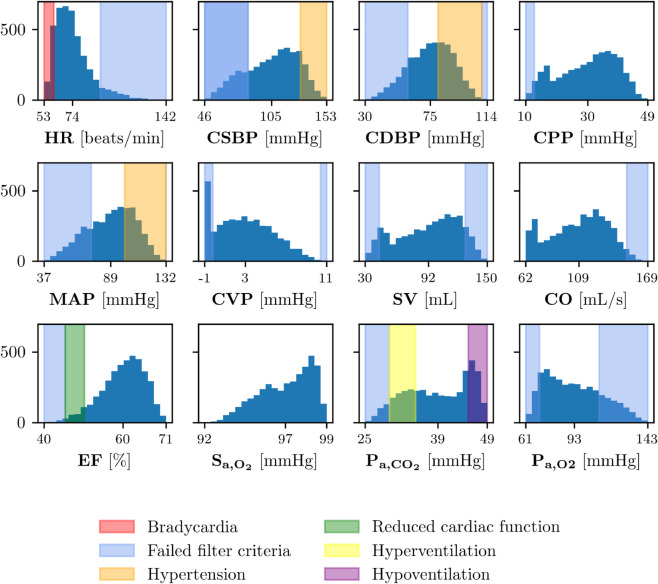
Virtual population distributions. Frequency (y-axes) of values of all model outputs (x-axes) for VP 1. The minimum, the mean, and the maximum of each variable are shown in the x-axes. Translucent blue bands indicate that the variable does not meet the filter criteria; translucent colored bands (red, orange, green, yellow, and purple) refer to the different CVRD. Abbreviations: HR: heart rate; CSBP/CDBP: central systolic/diastolic blood pressure; CPP: central pulse pressure; MAP: mean arterial pressure; CVP: central venous pressure; SV: left ventricle stroke volume; CO: left ventricle cardiac output; EF: left ventricle ejection fraction; S_a,O_2__: arterial O_2_ saturation; P_a,O_2__: arterial partial pressure of O_2_; P_a,CO_2__: arterial partial pressure of CO_2_.

**Fig 7:**
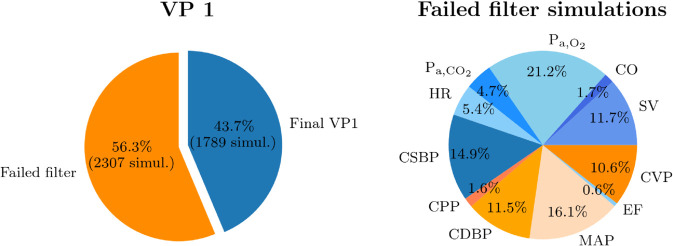
Post-processing filter criteria. On the left, the simulations that have failed the post-processing filter criteria among all 4096 simulations of VP 1. On the right, the percentage of failed simulations, for each output variable, among the simulations that have failed the filter criteria of VP 1. Abbreviations: HR: heart rate; CSBP/CDBP: central systolic/diastolic blood pressure; CPP: central pulse pressure; MAP: mean arterial pressure; CVP: central venous pressure; SV: left ventricle stroke volume; CO: left ventricle cardiac output; EF: left ventricle ejection fraction; S_a,O_2__: arterial O_2_ saturation; P_a,O_2__: arterial partial pressure of O_2_; P_a,CO_2__: arterial partial pressure of CO_2_.

Fig 6 shows the distributions of the model outputs of VP 1 generated with 4096 sampling points. The translucent blue bands over the histograms indicate that the value of that specific variable did not meet the filter criteria detailed in Table 3. The simulations with output variables falling within those regions were excluded from the VP. The other translucent colored bands (red, orange, green, yellow, and purple) refer to the different CVRD according to the cutoff values reported in Table 4. The shapes of the distributions vary depending on the physiological variable, with some showing clear peaks (e.g., HR, EF), while others exhibit more uniform or flat patterns (e.g., CPP, SV, CO). All variables are well distributed within their respective reference ranges, with the exception of S_a,O_2__, where a broader range would have been preferable. Indeed, the filter criteria are not applied to all variables of VP 1, particularly for S_a,O_2__, where the cutoff values in Table 3 are never met. Similarly, upper cutoff values for CSBP, CPP, MAP, EF and P_a,CO_2__ were never reached in pre-filtered VP1 subjects. In particular, Fig 7 shows, on the left, a pie chart illustrating which simulations failed the post-processing filter critera out of all 4096 simulations. On the right, a second pie chart shows the percentage of simulations that failed the filter criterion for each output variable, among the simulations that failed the filter criteria. Out of 4096 simulations, 2307 failed the filter criteria, therefore the final VP 1 is composed of 1789 subjects. Additionally, we note that the majority of failed simulations are due to too high P_a,O_2__, too low MAP, CSBP or CDBP, or too low CVP. Furthermore, it has been verified that a large portion of rejected samples is due to incompatible combinations of input parameters. For instance, a low value of $V_{tot}$ combined with a high value of $V_{u,ven}$ leads to excessively low MAP, while the opposite case, a high value of $V_{tot}$ combined with a low value of $V_{u,ven}$, results in an excessively high SV.

**Fig 8:**
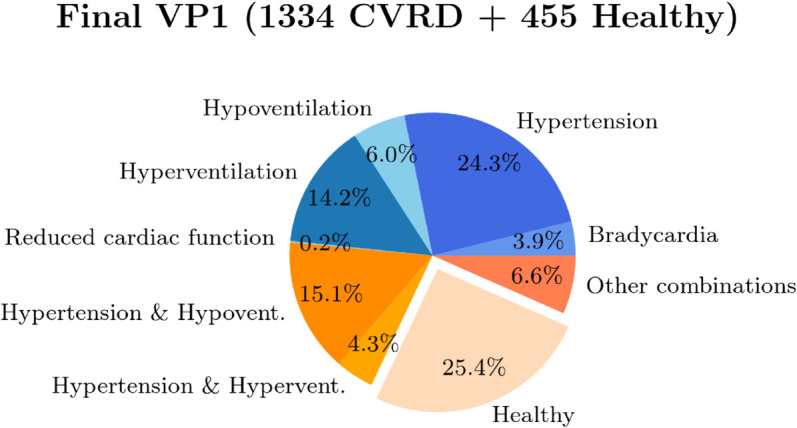
CVRD classification. CVRD classifications among the simulations that have not failed the filter criteria of VP 1.

Fig 8 shows the pie-chart of the CVRD classification among the simulations that have not failed the filter criteria. Among those, we can distinguish between healthy subjects (25.4 %), patients with bradycardia (3.9 %), hypertensive patients (24.3 %), hyper- or hypo-ventilating ones (14.2 % and 6.0 % respectively), patients with reduced cardiac function (0.2 %) or combinations of those.

Overall, the distribution ranges of the variables ensure a good level of heterogeneity, contributing to a diverse and realistic representation of the selected outputs. This allowed us to simulate a variety of scenarios, including a sufficient spectrum of pathological conditions.

**Fig 9:**
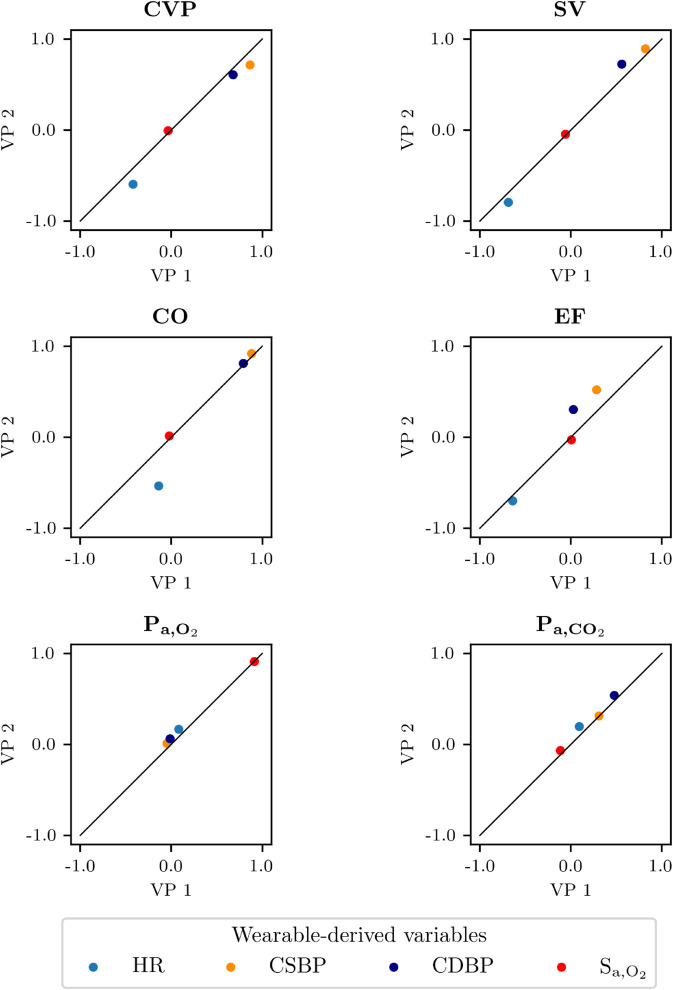
Wearable-derived and in-hospital variables correlations. Pearson correlation coefficients between the wearable-derived signals (HR, CSBP, CDBP, S_a,O_2__) and the in-hospital ones for VP1 (x axis) and VP2 (y axis). Abbreviations: HR: heart rate; CSBP/CDBP: central systolic/diastolic blood pressure; S_a,O_2__: arterial O_2_ saturation; CVP: central venous pressure; SV: left ventricle stroke volume; CO: left ventricle cardiac output; EF: left ventricle ejection fraction; P_a,O_2__: arterial partial pressure of O_2_; P_a,CO_2__: arterial partial pressure of CO_2_.

Lastly, we report a quantitative comparison between the final VPs, 1 and 2, after applying the filter criteria. Of the 4096 simulations of VP 2, 1336 samples were accepted after applying the filtering criteria specified in Table 3. Table 7 compares the final VPs in terms of means, SDs, minimums, and maximums. We observe that the descriptive statistics are similar across all variables in both populations, with minor variations due to differences in the parameter selection used for their generation. Fig 9 displays the Pearson correlation coefficients between the four wearable-derived signals and all the in-hospital variables for both final VPs. We can observe that, for most in-hospital variables, the differences in the correlation coefficients between the two VPs were negligible. However, the plots for CO and EF in Fig 9 highlight stronger correlations between HR and CO, and blood pressures and EF, in VP 2, compared with VP 1.

**Table 7:** Comparison between the final VPs 1 and 2.

Variable	Mean		SD		Min		Max		Unit
	VP 1	VP 2	VP 1	VP 2	VP 1	VP 2	VP 1	VP 2	
HR	69.5	69.9	7.8	5.7	55.2	59.9	93.9	92.4	$beats\cdot {min}^{-1}$
CSBP	112.5	111.0	13.4	13.3	84.1	84.3	145.5	140.4	mmHg
CDBP	79.8	79.2	9.8	8.9	59.1	60.3	107.1	101.2	mmHg
CPP	32.8	31.8	5.5	5.5	17.5	17.6	42.3	41.7	mmHg
MAP	95.5	94.5	11.4	10.9	74.1	74.0	125.7	120.1	mmHg
CVP	3.5	3.4	1.6	1.8	0.0	0.0	7.7	9.3	mmHg
SV	101.3	98.3	17.7	17.7	53.1	52.7	128.6	128.3	mL
CO	115.9	113.2	15.6	14.9	76.3	75.8	150.4	149.9	mL ⋅ s^−1^
EF	61.4	61.0	3.9	3.6	48.4	49.4	69.9	69.6	%
S_a,O_2__	96.3	96.3	1.2	1.2	93.1	92.3	98.5	98.4	mmHg
P_a,O_2__	87.9	88.7	11.6	11.6	70.0	70.0	110.0	110.0	mmHg
P_a,CO_2__	40.1	41.0	5.0	5.2	30.0	30.0	48.5	49.8	mmHg

Means, SDs, minimums and maximums of variables of interest of final VPs 1 and 2.. Abbreviations: HR: heart rate; CSBP/CDBP: central systolic/diastolic blood pressure; CPP: central pulse pressure; MAP: mean arterial pressure; CVP: central venous pressure; SV: left ventricle stroke volume; CO: left ventricle cardiac output; EF: left ventricle ejection fraction; S_a,O_2__: arterial O_2_ saturation; P_a,O_2__: arterial partial pressure of O_2_; P_a,CO_2__: arterial partial pressure of CO_2_.

### GPR models predictions.

**Fig 10:**
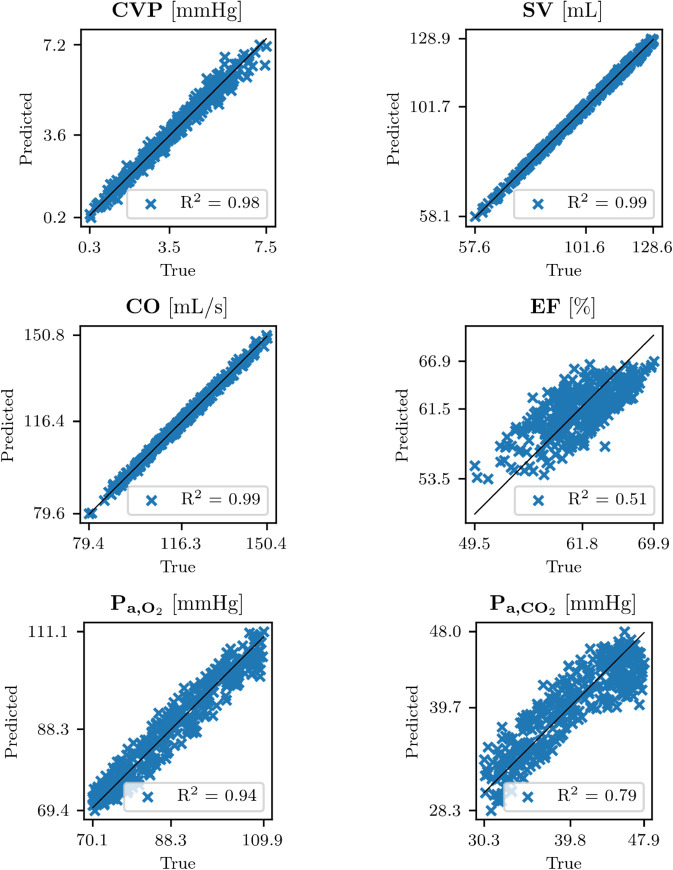
GPR models predictions versus true data. GPR models predictions (y-axes) versus true data (x-axes) for the final VP 1 test set, for all the in-hospital variables, together with the coefficient of determination $R^{2}$. Abbreviations: CVP: central venous pressure; SV: left ventricle stroke volume; CO: left ventricle cardiac output; EF: left ventricle ejection fraction; P_a,O_2__: arterial partial pressure of O_2_; P_a,CO_2__: arterial partial pressure of CO_2_.

Fig 10 shows GPR model predictions versus true data for the in-hospital variables on the final VP 1 test set (358 samples samples). The model demonstrated strong predictive performance across most variables, particularly for CVP, SV and CO. In contrast, the predictions for EF and the cardio-respiratory variables, i.e. P_a,O_2__ and P_a,CO_2__, were less precise, as indicated by lower R^2^ values. These findings align with the results in Fig 9, where the Pearson correlation coefficients for CVP, SV and CO were significantly higher in absolute value than those for EF, P_a,O_2__ and P_a,CO_2__.

Table 8 summarizes the GPR model predictions for perturbed and non-perturbed input features across the final VP 1 test set (358 samples) and the final VP 2 dataset (1336 samples) for all the in-hospital variables. We recall that the training of the GPR models was done only on the training set of the final VP 1 (1431 samples). For non-perturbed inputs, the table reports the following metrics: R^2^, maxRE (Eq 7), and MRE (Eq 8). For perturbed inputs, the table includes $maxRE_{PI}$ (Eq 13), $MRE_{PI}$ (Eq 14), as well as the means and SDs of **CV** (Eq 15).

**Table 8:** Performance of the GPR predictions.

Target	Test set	Without perturbed inputs			With perturbed inputs		
		R^2^	maxRE	MRE	$maxRE_{PI}$	$MRE_{PI}$	CV (mean ± SD)
CVP	VP 1	0.98	42.5 % (1.0 )	5.8 % (0.2 )	41.4 % (1.1 )	6.2 % (0.2 )	37.6 ± 24.0 %
	VP 2	0.50	889.7 % (4.1 )	39.4 % (1.0 )	929.6 % (4.1 )	39.8 % (1.0 )	51.2 ± 151.6 %
SV	VP 1	0.99	1.8 %	0.6 %	1.9 %	0.6 %	8.7 ± 1.7 %
	VP 2	0.99	1.8 %	0.6 %	2.1 %	0.6 %	9.1 ± 1.8 %
CO	VP 1	0.99	2.5 %	0.7 %	2.4 %	0.7 %	9.5 ± 1.6 %
	VP 2	0.99	1.8 %	0.6 %	2.0 %	0.6 %	9.8 ± 1.7 %
EF	VP 1	0.51	11.9 %	3.8 %	12.3 %	3.9 %	3.2 ± 1.0 %
	VP 2	0.68	8.8 %	2.9 %	11.9 %	2.9 %	3.0 ± 0.8 %
P_a,O_2__	VP 1	0.94	7.8 %	2.7 %	10.8 %	3.2 %	12.8 ± 3.2 %
	VP 2	0.94	9.7 %	2.7 %	9.8 %	3.1 %	12.6 ± 3.1 %
P_a,CO_2__	VP 1	0.79	16.4 %	4.8 %	19.5 %	5.1 %	14.3 ± 4.6 %
	VP 2	0.87	14.1 %	3.5 %	16.9 %	4.4 %	13.0 ± 3.4 %

Metrics and scoring of the GPR models predictions, with and without perturbed input data, on both the final VPs 1 and 2. Abbreviations: R^2^: coefficient of determination; maxRE: percentage maximum relative error of the GPR models predictions on test set; MRE: percentage mean relative error of the GPR models predictions on test set; $maxRE_{PI}$: percentage maximum relative error of the stochastic distribution of GPR models predictions with perturbed inputs on test set; $MRE_{PI}$: percentage mean relative error of the stochastic distribution of GPR models predictions with perturbed inputs on test set; CV: coefficient of variation of the stochastic distribution of GPR models predictions with perturbed inputs on test set; CVP: central venous pressure; SV: left ventricle stroke volume; CO: left ventricle cardiac output; EF: left ventricle ejection fraction; P_a,O_2__: arterial partial pressure of O_2_; P_a,CO_2__: arterial partial pressure of CO_2_. Unit:   = mmHg.

When comparing the GPR models performance between the final VPs 1 and 2, as reported in Table 8, negligible differences were observed for SV, CO and P_a,O_2__. Slightly larger differences were noted for EF and P_a,CO_2__, although the overall performance remains comparable. In contrast, the GPR model performance for CVP was significantly worse on the final VP 2. This is consistent with the fact that CVP exhibits high variability across the virtual patients in both VPs, with a standard deviation of ± 46% in VP 1 and ± 53% in VP 2 (see Table 7). The input feature vector of VP 1 used for the training of the GPR model is likely to lack some relevant information required to accurately predict CVP in the new test set (VP 2).

**Fig 11:**
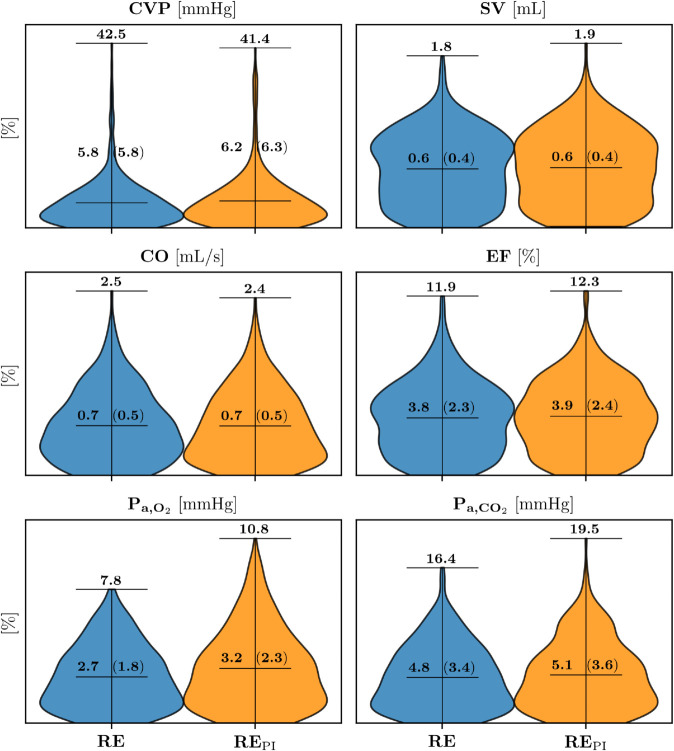
Relative errors of the GPR models predictions. Relative errors between the GPR models predictions and the true data of the in-hospital variables, with and without perturbed inputs (orange and blue distributions respectively), for the final VP 1 test set. Over each distributions we annotate the mean and the SD, between round brackets, of the error, together with the maximum error, at the top of each distribution. Abbreviations: RE: percentage relative error of the GPR models predictions without perturbed inputs; $RE_{PI}$: percentage relative error of the stochastic distribution of GPR models predictions with perturbed inputs; CVP: central venous pressure; SV: left ventricle stroke volume; CO: left ventricle cardiac output; EF: left ventricle ejection fraction; P_a,O_2__: arterial partial pressure of O_2_; P_a,CO_2__: arterial partial pressure of CO_2_

When comparing the performance of the GPR models with perturbed and non-perturbed input data, as reported in Table 8, we observed negligible differences in the results. This is further illustrated in Fig 11, which shows the relative errors for the GPR models across all predicted variables in the final VP 1 test set. The figure compares the distributions of **RE** (relative errors for non-perturbed data, shown in blue) and ${RE}_{PI}$ (relative errors for perturbed data, shown in orange). For each distribution, the means and SDs are displayed above the mean lines, while the maximum relative errors are indicated at the top. These plots demonstrate that the performance of the GPR models remains consistent between perturbed and non-perturbed input data, indicating that the method remains robust and performs well even when physiological perturbations are applied to the input data.

**Fig 12:**
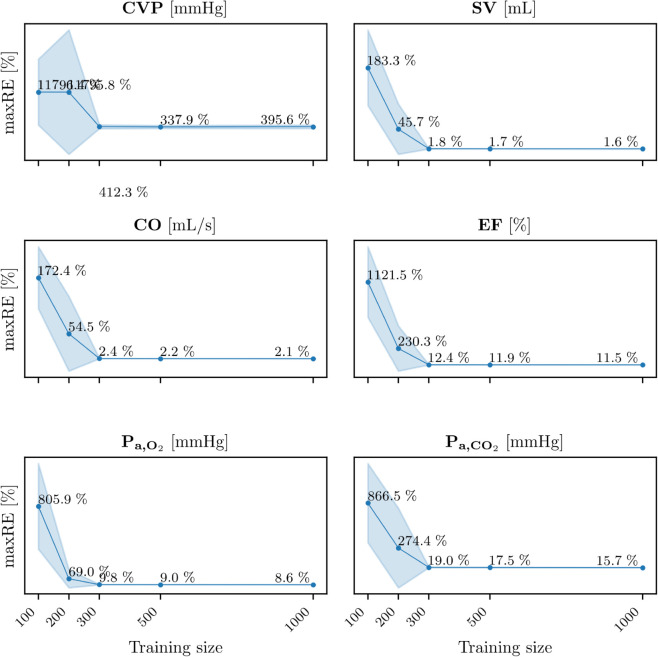
Learning curves of the GPR models. Learning curves of the percentage maximum relative errors of the GPR models predictions without perturbed input data (y-axes) for 100, 200, 300, 500 and 1000 training sizes sampled from the final VP 1 training set (x-axes). Abbreviations: maxRE: percentage maximum relative error of the GPR models predictions without perturbed inputs; CVP: central venous pressure; SV: left ventricle stroke volume; CO: left ventricle cardiac output; EF: left ventricle ejection fraction; P_a,O_2__: arterial partial pressure of O_2_; P_a,CO_2__: arterial partial pressure of CO_2_.

Fig 12 shows the learning curves of maxRE for all GPR models trained with 100, 200, 300, 500, and 1000 samples drawn from the final VP 1 training set. The results indicate that the performance of the GPR models improves significantly with a training set size of at least 300 samples, suggesting that a minimum of 300 training samples is required to ensure reliable model performance.

## Discussion

### Virtual database

In this study, we presented a methodology for generating a VP dataset that includes both healthy individuals and patients with CVRD, such as hypertensive patients, hyper- or hypo-ventilating ones, or combinations of those (see Fig 8). This was achieved using an advanced computational cardio-respiratory model capable of simulating a wide range of clinically relevant variables. Although there are existing studies focused on generating virtual populations [17,41,42,78,79], to the best of our knowledge, our dataset is the most comprehensive in terms of the number of features it includes, spanning both cardiovascular and respiratory characteristics.

One of the key strengths of our approach is its speed and efficiency, in terms of time and computational cost. One simulation runs in 3 min and 45 s on a system equipped with an AMD^®^ Ryzen^TM^ Threadripper 3990X processor, featuring 64 cores and 128 threads, which allowed us to generate up to 5000 virtual patients in approximately 15 hours, with 20 simulations running concurrently. This ability to quickly generate vast datasets opens up new opportunities for predictive modeling, particularly when real patient data may not be available in sufficient quantity or quality.

Clinical datasets often predominantly consist of patients who are hospitalized, typically under the effect of drugs, or undergoing surgery, thus in advanced stages of disease [11–14,21,38]. This makes it challenging to study predictive models for a healthy population or individuals with less acute conditions, such as those with early-stage forms of disease. In contrast, our dataset specifically describes these conditions, including healthy states and mild to moderate CVRD.

Moreover, our VP generation methodology could be used in the future to simulate a broader variety of CVRD, expanding the selection and refinement of parameter ranges and their combinations. This could also include wave propagation models for the circulatory system, such as those in [80,81], which would allow addressing additional pathological aspects and enable more targeted predictive studies, as in [41,78,79]. This would not only allow for the inclusion of additional conditions but also enable the modeling of different levels of severity within specific diseases, thus providing a more nuanced dataset for predictive studies. By designing and simulating more targeted scenarios, this approach could enhance the development of more accurate, robust, and clinically relevant predictive models.

### Prediction of in-hospital variables

This study demonstrates the potential of using virtual patient data to predict in-hospital variables, such as CVP, CO, SV, EF, P_a,O_2__ and P_a,CO_2__, from signals that are acquirable through wearable devices, i.e. HR, BPs and S_a,O_2__. In this in silico context, GPR emerges as a particularly suitable predictive methodology. Importantly, these predictions show good accuracy, even when accounting for potential errors introduced by wearable signal acquisition, as shown in Fig 11. Specifically (see Table 8), the mean relative errors for non-perturbed data are as follows: CVP: 5.8 % (0.2 mmHg), SV: 0.6 %, CO: 0.7 %, EF: 3.8 %, P_a,O_2__: 2.7 %, and P_a,CO_2__: 4.8 %. When comparing these results to those obtained with perturbed inputs, the differences were negligible: CVP: 6.2 % (0.2 mmHg), SV: 0.6 %, CO: 0.7 %, EF: 3.9 %, P_a,O_2__: 3.2 %, and P_a,CO_2__: 5.1 %. Cardiovascular parameters like CVP, SV and CO, show consistently high accuracy. Whereas, the predictions for EF, P_a,O_2__ and P_a,CO_2__ were slightly less precise but still demonstrate good accuracy.

In terms of clinical acceptability, there is no universal definition of error thresholds, as they vary depending on the variable being measured and the methodology in use. However, considering that wearable devices primarily serve as screening tools rather than diagnostic instruments, a certain degree of error is generally tolerated. Within this context, the error thresholds observed in our study can be considered clinically acceptable, as they align with the intended purpose of these devices in preliminary assessment and continuous monitoring.

Moreover, to ensure that the predictive capabilities of our model are not overly dependent on the specific parameter choices that we made for the generation of the VP, we tested the GPR models, which were trained on the VP 1 dataset, on a new dataset, VP 2, obtained varying different combinations of parameters. In particular, the mean relative error differences between the performance on VP 1 and VP 2, for the variables predicted with perturbed input, are (see Table 8): SV (0.0 %), CO (0.1 %), EF (1.0 %), P_a,O_2__ (0.1 %), followed by P_a,CO_2__ (0.7 %), and finally CVP (0.8 mmHg). For all the predicted variables, with the exception of CVP, the mean relative error differences are negligible. These results suggest that the model’s predictive performance is not overly reliant on the specific characteristics of the dataset used for training, which provides confidence that the model can be effectively applied to different, potentially real-world, datasets.

One of the critical factors enabling the high accuracy of predictions in this study is the large dataset of virtual patients generated through our computational framework. Results reported in section GPR models predictions revealed that at least 300 training samples are required to achieve satisfactory predictions. However, generating such a large dataset using real-world signals is often time-consuming, resource-intensive, or even infeasible due to logistical and ethical constraints. To address this limitation, future research could explore the combination of real-world data with in silico generated data for training GPR models, as explored in [82,83]. By augmenting real patient datasets with virtual data, it might be possible to develop predictive models that perform well on real-world data while reducing the dependence on extensive real datasets. This hybrid approach could facilitate the creation of accurate and clinically relevant predictive tools, particularly in scenarios where data collection is challenging or constrained.

### Limitations

The results presented in this study were obtained entirely in an in silico setting. Although the computational framework and generated datasets have been rigorously tested, real-world validation is necessary to confirm the clinical utility and reliability of the predictive models.

In the local sensitivity analysis performed on the cardio-respiratory model, each parameter is varied by 20 % to assess its impact on the clinically relevant variables. However, this approach does not account for the fact that some parameters may exhibit greater or smaller physiological variability. Assigning physiologically accurate ranges to each model parameter is challenging, particularly considering the large number of parameters involved (286). Additionally, the local sensitivity analysis does not capture potential interactions between parameters, which could also influence the model’s outputs. Therefore, while the local sensitivity analysis provides a computationally efficient way to assess individual parameter impacts, a more comprehensive approach would involve performing a global sensitivity analysis, and a more refined selection of parameter variability ranges. This would ultimately lead to a more accurate choice of parameters for the generation of the VP. However, such approach would demand significantly more computational resources and an optimized methodology to remain feasible.

The virtual dataset is generated by varying the most significant model parameters within physiologically plausible ranges and, after applying the filter criteria, all virtual variables align with physiological or pathological ranges. However, the cardio-respiratory model has not been specifically parameterized to reproduce full pathological profiles. Instead, virtual subjects were identified as pathological based solely on certain output variables exceeding literature-reported threshold values. Consequently, we have not verified whether their overall physiological profile was consistent with that of a real patient.

### Conclusion

This study highlights the potential for predictive studies on remote and continuous monitoring of both healthy individuals and those affected by cardiovascular and respiratory diseases, using virtual patient datasets generated through an advanced computational cardio-respiratory model. The ability to simulate a broad range of physiological conditions, provides a resource for developing and testing predictive tools, especially in scenarios where real-world data is limited or unavailable. A critical advantage of this methodology is its ability to generate large, diverse datasets in a computationally efficient manner, facilitating the development of predictive models that require substantial training data.

The results demonstrate the high accuracy of GPR-based predictions for key cardiovascular and respiratory parameters, even under conditions of perturbed input data. Moreover, the negligible differences observed when testing models trained on one virtual patient dataset against a second independently generated dataset, indicate that the predictive capabilities are not overly dependent on the specific parameter choices used to generate the virtual population. This suggests that the proposed approach is generalizable across different datasets.

Future efforts should focus on refining the parameter ranges and combinations to expand the variety of simulated conditions, as well as on testing the methodology on real patient data, exploring also the possibility of an hybrid approach with both real and in silico data.

## Supporting information

S1 Appendix Cardio-respiratory model equations and validation. The model is a non-linear system of differential-algebraic equations describing cardiovascular functions, including blood circulation, respiration, gas transport, and short-term regulation.
(PDF)

S2 Appendix Definition of cardiovascular and cardio-respiratory indexes. A precise description of the computation of the extracted model output variables.
(PDF)

S3 Appendix Virtual population 2 results. Convergence, distributions, post-processing filter and CVRD-classification.
(PDF)
