# Supplementary material for: Predicting in-hospital indicators from wearable-derived signals for cardiovascular and respiratory disease monitoring: An in silico study
Source: PLOS Digit Health. 2025 Oct 14;4(10):e0001041. doi: 10.1371/journal.pdig.0001041 (PMC12520384; doi:10.1371/journal.pdig.0001041)
Supplement: S1 Appendix — The model is a non-linear system of differential-algebraic equations describing cardiovascular functions, including blood circulation, respiration, gas transport, and short-term regulation. (PDF) [file pdig.0001041.s001.pdf]

Supplementary Material for: Predicting in-hospital indicators from wearable-derived signals for cardiovascular and respiratory disease monitoring: an in silico study, by Bianca Maria Laudenzi, Alberto Cucino, Sergio Lassola, Eleonora Balzani, Lucas Omar Müller. Corresponding author: Bianca Maria Laudenzi, biancamaria.laudenzi@unitn.it

Cardio-respiratory model equations and validation.

The model comprises a nonlinear system of Differential-Algebraic Equations that characterize cardiovascular functions, including blood flow in the heart chambers, systemic and pulmonary circulation, respiration, gas transport, metabolism, and primary short-term regulatory mechanisms. The equations presented here are obtained following the framework presented in Albanese et al.[2] with some modifications.

The circulatory system

The equations describing the circulatory system have been obtained by enforcing conservation of mass and balance of forces for each vascular compartment as in [2]. The circulatory system comprises the systemic and the pulmonary circulations, and the heart, with the four cardiac chambers and the cardiac valves.

Systemic circulation

The systemic circulation system comprises the systemic arteries, the peripheral arteries and veins, divided in splanchnic peripheral compartment, extra-splanchnic peripheral compartment, skeletal muscle peripheral compartment, brain peripheral compartment and coronary peripheral compartment, and the thoracic vein. In this section the model output variables are denotes as V which represents the blood volume (measured in mL), q which denotes the blood flow rate (expressed in mL/s), and P that stands for pressure (measured in mmHg). Note that the peripheral resistances R_{jp} with $j = \{\mathrm{s,e,m,b,h}\}$ (measured in mmHg s/mL) and the unstressed volumes, $V_{\mathrm{u,sv}}$, $V_{\mathrm{u,mv}}$ and $V_{\mathrm{u,ev}}$ (measured in mL), are as well model output variables since they can be modified by the sympathetic efferent pathways of the cardiovascular control (see section Cardiovascular control models). The assignment of the parameters is based on [2] when possible, otherwise the parameters were calibrated so as to fit reference literature output variables (see Table 9 for main model-predicted cardiovascular and respiratory indexes and reference values reported in the literature). The values of the parameters appearing in equations (1)-(19) are reported in Table 1.

Variables subscripts: sa, systemic arteries; AV, aortic valve compartment; sp, splanchnic peripheral arteries; ep, extra-splanchnic peripheral arteries; mp, skeletal muscle peripheral arteries; bp, brain peripheral arteries; hp, coronary peripheral arteries; sv, splanchnic peripheral veins; ev, extra-splanchnic peripheral veins; mv, skeletal muscle peripheral veins; bv, brain peripheral veins; hp, coronary peripheral veins; tv, thoracic veins; RA, right atrium; rv, right ventrivle; pl, pleural space; tm, transmural.

Systemic arteries

$$\frac{dV_{\mathrm{sa}}}{dt} = q_{\mathrm{AV}} - q_{\mathrm{sa}}, \tag{1}$$

$$q_{\mathrm{sa}} = \sum_j q_{\mathrm{jp,in}}, \qquad \text{with } j = \{\mathrm{s,e,m,b,h}\}, \tag{2}$$

$$q_{\mathrm{jp,in}} = \frac{P_{\mathrm{sa}} - P_{\mathrm{jp}}}{R_{\mathrm{p}}}, \tag{3}$$

$$P_{\mathrm{sa}} = \frac{V_{\mathrm{sa}} - V_{\mathrm{u,sa}}}{C_{\mathrm{sa}}}. \tag{4}$$

$$\tag{5}$$

Peripheral arteries

$$\frac{dV_{\rm jp}}{dt} = q_{\rm jp,in} - q_{\rm jp}, \qquad \text{with } \ j = \{\rm s, e, m, b, h\}, \tag{6}$$

$$q_{\rm jp} = \max\left(0, \frac{P_{\rm jp} - P_{\rm jv}}{R_{\rm jp}}\right), \tag{7}$$

$$P_{\rm jp} = \frac{V_{\rm jp} - V_{\rm u,jp}}{C_{\rm jp}}. \tag{8}$$

$$\tag{9}$$

Peripheral veins

$$\frac{dV_{\rm jv}}{dt} = q_{\rm jp} - q_{\rm jv}, \qquad \text{with } \ j = \{\rm s, e, m, b, h\}, \tag{10}$$

$$P_{\rm ev} = \frac{\max\left(0, V_{\rm ev} - V_{\rm u,ev}\right)}{C_{\rm ev}}, \tag{11}$$

$$P_{\rm jv} = \frac{V_{\rm jv} - V_{\rm u,jv}}{C_{\rm jv}}, \tag{12}$$

$$q_{\rm jv} = \max\left(0, \frac{P_{\rm jv} - P_{\rm tv}}{R_{\rm jv}}\right). \tag{13}$$

Thoracic vein

$$\frac{dV_{\rm tv}}{dt} = \sum_j q_{\rm jv} - q_{\rm tv}, \qquad \text{with } \ j = \{\rm s, e, m, b, h\}, \tag{14}$$

$$q_{\rm tv} = \max\left(\frac{P_{\rm tv} - P_{\rm RA}}{R_{\rm tv}}, 0\right), \tag{15}$$

$$P_{\rm tv} = P_{\rm pl} + P_{\rm tm,tv}, \tag{16}$$

$$P_{\rm tm,tv} = \begin{cases} D_1 + K_1(V_{\rm tv} - V_{\rm u,tv}) - \psi, & \text{if } \ V_{\rm tv} \geq V_{\rm u,tv}, \\ D_2 + K_2\, e^{\frac{V_{\rm tv}}{V_{\rm tv,min}}} - \psi, & \text{if } \ V_{\rm tv} < V_{\rm u,tv}, \end{cases} \tag{17}$$

$$\psi = \frac{K_{\rm xp}}{e^{\frac{V_{\rm tv}}{K_{\rm xv}}-1}}, \tag{18}$$

$$R_{\rm tv} = K_{\rm R}\left(\frac{V_{\rm tv,max}}{V_{\rm tv}}.\right) + R_{\rm tv0}. \tag{19}$$

Pulmonary circulation

The pulmonary circulation system comprises the pulmonary arteries, the peripheral arteries, placed in parallel with the pulmonary shunt, and the pulmonary veins. Here, the model output variables are represented as follows: V, which indicates blood volume (measured in mL); q, which represents the blood flow rate (expressed in mL/s); and P, which denotes pressure (measured in mmHg). The values of the parameters in equations (20)-(36) are reported in Table 1.

Variables subscripts: pa, pulmonary arteries; PV, pulmonary valve; pp, peripheral pulmonary arteries; ps, pulmonary shunt; pv, pulmonary veins; pl, pleural space.

Pulmonary arteries

$$\frac{dV_{\mathrm{pa}}}{dt} = q_{\mathrm{PV}} - q_{\mathrm{pa}}, \tag{20}$$

$$q_{\mathrm{pa}} = \sum_j q_{\mathrm{pj,in}}, \qquad \text{with } j = \{\mathrm{s}, \mathrm{p}\}, \tag{21}$$

$$q_{\mathrm{pp,in}} = \frac{P_{\mathrm{pa}} - P_{\mathrm{pp}}}{R_{\mathrm{pp,in}}}, \tag{22}$$

$$q_{\mathrm{ps,in}} = \frac{P_{\mathrm{pa}} - P_{\mathrm{ps}}}{R_{\mathrm{ps,in}}}, \tag{23}$$

$$P_{\mathrm{pa}} = \frac{V_{\mathrm{pa}} - V_{\mathrm{u,pa}}}{C_{\mathrm{pa}}}. \tag{24}$$

$$\tag{25}$$

Peripheral arteries

$$\frac{dV_{\mathrm{pp}}}{dt} = q_{\mathrm{pp,in}} - q_{\mathrm{pp}}, \tag{26}$$

$$\frac{dV_{\mathrm{ps}}}{dt} = q_{\mathrm{ps,in}} - q_{\mathrm{ps}}, \tag{27}$$

$$q_{\mathrm{pp}} = \frac{P_{\mathrm{pp}} - P_{\mathrm{pv}}}{R_{\mathrm{pp}} \frac{100}{(100 - sh)}}, \tag{28}$$

$$q_{\mathrm{ps}} = \frac{P_{\mathrm{ps}} - P_{\mathrm{pv}}}{R_{\mathrm{pp}} \frac{100}{sh}}, \tag{29}$$

$$P_{\mathrm{pp}} = \left(V_{\mathrm{pp}} - V_{\mathrm{u,pp}} \frac{(100 - sh)}{100} \right) \frac{100}{C_{\mathrm{pp}}(100 - sh)} + P_{\mathrm{pl}}, \tag{30}$$

$$P_{\mathrm{ps}} = \left(V_{\mathrm{ps}} - V_{\mathrm{u,pp}} \frac{sh}{100} \right) \frac{100}{C_{\mathrm{pp}} sh} + P_{\mathrm{pl}}. \tag{31}$$

$$\tag{32}$$

Peripheral veins

$$\frac{dV_{\mathrm{pv}}}{dt} = q_{\mathrm{pp}} + q_{\mathrm{ps}} - q_{\mathrm{pv}}, \tag{33}$$

$$q_{\mathrm{pv}} = \max\left(0, \frac{P_{\mathrm{pv}} - P_{\mathrm{la}}}{R_{\mathrm{pv}}}\right), \tag{34}$$

$$P_{\mathrm{pv}} = \frac{V_{\mathrm{pv}} - V_{\mathrm{u,pv}}}{C_{\mathrm{pv}}} + P_{\mathrm{pl}}. \tag{35}$$

$$\tag{36}$$

Heart

The heart compartment comprises the cardiac valves, the ventricles and the atria. Flow through all valves is governed by the valve model proposed in [14]. Heart chambers are described as time-varying elastances, with prescribed non-dimensional elastance functions, defined as in [10] for the atria, whereas as in [17] for the ventricles. In this section, the model output variables are represented as follows: V indicates blood volume (measured in mL); q represents the blood flow rate (expressed in mL/s); P denotes pressure (measured in mmHg), B represents the Bernoulli's resistance (expressed in g/mL/cm), L denotes the blood inertance (expressed in g/mL/cm^4); A^{eff} indicates the effective area of the cardiac valves (measured in cm^2); ζ_v represents the opening state of valves (which is non-dimensional, ranging from 0 to 1); E is the heart chambers time-varying elastance (expressed in mmHg/mL); e is the heart chambers time-varying elastance activation function (which is non-dimensional, ranging from 0 to 1); t is the time (measured in s); $R_{\mathcal{V}}$ is the viscous resistance of the ventricles (measured in mmHg s/mL); u represents the fraction of cardiac cycle (which is dimensionless, ranging from 0 to 1); T_{sys} is the

duration of systole (expressed in s); T is the cardiac cycle duration (expressed in s). Note that the cardiac cycle T and the active elastances of the ventricles $E_{\mathrm{LV,max}}$ and $E_{\mathrm{RV,max}}$ are model output variables since they can be modified by the cardiovalscular control model (see section Cardiovascular control models). The values of the parameters appearing in equations (37)-(52) are taken from [14] and are reported in Table 2. The values of the parameters appearing in equations (53)-(65) are taken from [10] and from [17], and are reported in Table 3.

Variables subscripts: TV, tricuspid valve; PV, pulmonary valve; MV, mitral valve; AV, aortic valve; LA, left atrium; RA, right atrium; LV, left ventricle; RV, right ventricle; pv; pulmonary veins; tv; thoracic veins; pl, pleural space.

Cardiac valves

$$\frac{dq_{\mathrm{TV}}}{dt} = \frac{1}{L_{\mathrm{TV}}}\left(\Delta P_{\mathrm{TV}} - B_{\mathrm{TV}} q_{\mathrm{TV}} \left|q_{\mathrm{TV}}\right|\right), \tag{37}$$

$$\Delta P_{\mathrm{TV}} = P_{\mathrm{RA}} - P_{\mathrm{RV}}. \tag{38}$$

$$\frac{dq_{\mathrm{PV}}}{dt} = \frac{1}{L_{\mathrm{PV}}}\left(\Delta P_{\mathrm{PV}} - B_{\mathrm{PV}} q_{\mathrm{PV}} \left|q_{\mathrm{PV}}\right|\right), \tag{39}$$

$$\Delta P_{\mathrm{PV}} = P_{\mathrm{RV}} - \Delta P^{\mathrm{P}}_{\mathrm{prox}}, \tag{40}$$

$$\Delta P^{\mathrm{P}}_{\mathrm{prox}} = P_{pa} + R^{\mathrm{P}}_{\mathrm{prox}} q_{\mathrm{PV}}. \tag{41}$$

$$\frac{dq_{\mathrm{MV}}}{dt} = \frac{1}{L_{\mathrm{MV}}}\left(\Delta P_{\mathrm{MV}} - B_{\mathrm{MV}} q_{\mathrm{MV}} \left|q_{\mathrm{MV}}\right|\right), \tag{42}$$

$$\Delta P_{\mathrm{MV}} = P_{\mathrm{LA}} - P_{\mathrm{LV}}. \tag{43}$$

$$\frac{dq_{\mathrm{AV}}}{dt} = \frac{1}{L_{\mathrm{AV}}}\left(\Delta P_{\mathrm{AV}} - B_{\mathrm{AV}} q_{\mathrm{AV}} \left|q_{\mathrm{AV}}\right|\right), \tag{44}$$

$$\Delta P_{\mathrm{AV}} = P_{\mathrm{LV}} - \Delta P^{\mathrm{S}}_{\mathrm{prox}}, \tag{45}$$

$$\Delta P^{\mathrm{S}}_{\mathrm{prox}} = P_{sa} + R^{\mathrm{S}}_{\mathrm{prox}} q_{\mathrm{AV}}, \tag{46}$$

$$B_v = \frac{\rho}{2\left(A_v^{\mathrm{eff}}\right)^2}, \qquad \text{with } v = \{\mathrm{TV}, \mathrm{PV}, \mathrm{MV}, \mathrm{AV}\}, \tag{47}$$

$$L_v = \frac{\rho l_v^{\mathrm{eff}}}{A_v^{\mathrm{eff}}}, \tag{48}$$

$$A_v^{\mathrm{eff}} = \left(A_v^{\mathrm{eff,max}} - A_v^{\mathrm{eff,min}}\right)\zeta_v + A_v^{\mathrm{eff,\ min}}\ , \tag{49}$$

$$A_v^{\mathrm{eff,min}} = M^v_{\mathrm{rg}} A^v_{\mathrm{ann}}, \tag{50}$$

$$A_v^{\mathrm{eff,max}} = M^v_{\mathrm{st}} A^v_{\mathrm{ann}}, \tag{51}$$

$$\frac{d\zeta_v}{dt} = \begin{cases} \left(1-\zeta_v\right) K^v_{\mathrm{open}}\left(\Delta P_v - \Delta P^v_{\mathrm{open}}\right), & \text{if } \Delta P_v \geq \Delta P^v_{\mathrm{open}}, \\ 0, & \text{if } \Delta P^v_{\mathrm{close}}\ < \Delta P_v < \Delta P^v_{\mathrm{open}}, \\ \zeta v K^v_{\mathrm{close}}\ \left(\Delta P_v - \Delta P^v_{\mathrm{close}}\ \right), & \text{if } \Delta P_v \leq \Delta P_{\mathrm{close}}\ . \end{cases} \tag{52}$$

Heart chambers

$$\frac{dV_{\mathrm{LA}}}{dt} = q_{\mathrm{pv}} - q_{\mathrm{MV}}, \tag{53}$$

$$\frac{dV_{\mathrm{RA}}}{dt} = q_{\mathrm{tv}} - q_{\mathrm{TV}}, \tag{54}$$

$$\frac{dV_{\mathrm{LV}}}{dt} = q_{\mathrm{MV}} - q_{\mathrm{AV}}, \tag{55}$$

$$\frac{dV_{\mathrm{RV}}}{dt} = q_{\mathrm{TV}} - q_{\mathrm{PV}}. \tag{56}$$

$$P_{\mathcal{A}} = E_{\mathcal{A}}\left(V_{\mathcal{A}} - V_{\mathrm{u},\mathcal{A}}\right) + S\frac{dV_{\mathcal{A}}}{dt} + P_{\mathrm{pl}}, \qquad \text{with } \mathcal{A} = \{\mathrm{LA}, \mathrm{RA}\}, \tag{57}$$

$$E_{\mathcal{A}} = E^{\mathrm{A}}_{\mathcal{A}} e_{\mathcal{A}} + E^{\mathrm{B}}_{\mathcal{A}}, \tag{58}$$

$$e_{\mathcal{A}}(t) = \begin{cases} \frac{1}{2}\left\{1 + \cos\left[\pi\left(t + T - t^{\mathcal{A}}_{\mathrm{r}}\right)/T^{\mathcal{A}}_{\mathrm{rp}}\right]\right\}, & \text{if } 0 \le t \le t^{\mathcal{A}}_{\mathrm{r}} + T^{\mathcal{A}}_{\mathrm{rp}} - T, \\ 0, & \text{if } t^{\mathcal{A}}_{\mathrm{r}} + T^{\mathcal{A}}_{\mathrm{rp}} - T < t \le t^{\mathcal{A}}_{\mathrm{c}}, \\ \frac{1}{2}\left\{1 - \cos\left[\pi\left(t - t^{\mathcal{A}}_{\mathrm{c}}\right)/T^{\mathcal{A}}_{\mathrm{c}}\right]\right\}, & \text{if } t^{\mathcal{A}}_{\mathrm{c}} < t \le t^{\mathcal{A}}_{\mathrm{c}} + T^{\mathcal{A}}_{\mathrm{cp}}, \\ \frac{1}{2}\left\{1 + \cos\left[\pi\left(t - t^{\mathcal{A}}_{\mathrm{r}}\right)/T^{\mathcal{A}}_{\mathrm{rp}}\right]\right\}, & \text{if } t^{\mathcal{A}}_{\mathrm{c}} + T^{\mathcal{A}}_{\mathrm{cp}} < t \le T, \end{cases} \tag{59}$$

$$P_{\mathcal{V}} = P_{\mathcal{V},\max} - R_{\mathcal{V}} q_{\mathrm{AV}}, \qquad \text{with } \mathcal{V} = \{\mathrm{LV}, \mathrm{RV}\}, \tag{60}$$

$$R_{\mathcal{V}} = \max\left(0, k_{\mathrm{R},\mathcal{V}} P_{\mathcal{V},\max}\right), \tag{61}$$

$$P_{\mathcal{V},max} = e_{\mathcal{V}}(t) E_{\mathcal{V},\max}\left(V_{\mathcal{V}} - V_{\mathrm{u},\mathcal{V}}\right) + \left(1 - e_{\mathcal{V}}(t)\right) P_{0,\mathcal{V}}\left(e^{k_{\mathrm{E},\mathcal{V}} V_{\mathcal{V}}} - 1\right) + P_{pl}, \tag{62}$$

$$e_{\mathcal{V}}(t) = \begin{cases} \left[\sin\left(\frac{\pi T(t)}{T_{\mathrm{sys}}(t)} u\right)\right]^2, & \text{if } 0 \le u \le \frac{T_{\mathrm{sys}}}{T}, \\ 0, & \text{if } \frac{T_{\mathrm{sys}}}{T} \le u \le 1. \end{cases} \tag{63}$$

$$T_{\mathrm{sys}} = T_{\mathrm{sys},0} - k_{\mathrm{sys}}\frac{1}{T}, \tag{64}$$

$$\begin{cases} \frac{d\varepsilon}{dt} = \frac{1}{T(t)}, \\ u(t) = \mathrm{dec}(\varepsilon), \end{cases} \tag{65}$$

where $\mathrm{dec}(\cdot)$ is a function that takes the decimal part of a real positive number.

The lung mechanics

The equations describing the lung mechanics model have been obtained by applying the conservation of mass for each compartment and are taken from [2]. Linear resistances represents the dissipative forces that act two-by-two on larynx, trachea, bronchea, and alveoli during normal breathing. On the other hand, the linear compliancies describe the elastic forces. Here, the model variables are the following: V denotes the air volume (measured in mL); $\dot{V}$ denotes the airflow (expressed in mL/s); P stands for pressure (measured in $\mathrm{cmH_2O}$); T_{E} and T_{I} are respectively the expiratory and inspiratory periods (expressed in s); T_R is the respiratory cycle duration; RR is the respiratory rate (measured in breaths/min); and τ is the time constant that shapes the respiratory muscle pressure (measured in s); V_{D} is the dead space volume (measured in mL). Note that the $P_{\mathrm{mus,min}}$ and the RR are model variables since they can be modified by ventilatory control mechanisms (see section Respiratory control models). The values of the parameters appearing in equations (66)-(84) are taken from [2] and are reported in Table 4.

Variables subscripts: ao, airway opening; m, mouth; l, larynx; tr, trachea; b, bronchea; A, alveoli; pl, pleural space; mus; respiratory muscles.

Larinx

$$\frac{dV_{\mathrm{l}}}{dt} = \dot{V}_{\mathrm{ao,l}} - \dot{V}_{\mathrm{l,tr}}, \tag{66}$$

$$\dot{V}_{\mathrm{ao,l}} = \frac{P_{\mathrm{ao}} - P_{\mathrm{l}}}{R_{\mathrm{m,l}}}, \tag{67}$$

$$\dot{V}_{\mathrm{l,tr}} = \frac{P_{\mathrm{l}} - P_{\mathrm{tr}}}{R_{\mathrm{l,tr}}}, \tag{68}$$

$$P_{\mathrm{l}} = \frac{V_{\mathrm{l}} - V_{\mathrm{u,l}}}{C_{\mathrm{l}}}. \tag{69}$$

Trachea

$$\frac{dV_{\text{tr}}}{dt} = \dot{V}_{\text{l,tr}} - \dot{V}_{\text{tr,b}}, \tag{70}$$

$$\dot{V}_{\text{tr,b}} = \frac{P_{\text{tr}} - P_{\text{b}}}{R_{\text{tr,b}}}, \tag{71}$$

$$P_{\text{tr}} = \frac{V_{\text{tr}} - V_{\text{u,tr}}}{C_{\text{tr}}} + P_{\text{pl}}. \tag{72}$$

Bronchea

$$\frac{dV_{\text{b}}}{dt} = \dot{V}_{\text{tr,b}} - \dot{V}_{\text{b,A}}, \tag{73}$$

$$\dot{V}_{\text{b,A}} = \frac{P_{\text{b}} - P_{\text{A}}}{R_{\text{b,A}}}, \tag{74}$$

$$P_{\text{b}} = \frac{V_{\text{b}} - V_{\text{u,b}}}{C_{\text{b}}} + P_{\text{pl}}. \tag{75}$$

Alveoli

$$\frac{dV_{\text{A}}}{dt} = \dot{V}_{\text{b,A}}, \tag{76}$$

$$P_{\text{A}} = \frac{V_{\text{A}} - V_{\text{u,A}}}{C_{\text{A}}} + P_{\text{pl}}. \tag{77}$$

Pleural space

$$P_{\text{pl}} = \frac{1}{C_{\text{cw}}} V_{\text{pl,i}} + P_{\text{pl,EE}} + P_{\text{mus}}, \tag{78}$$

$$\frac{dV_{\text{pl,i}}}{dt} = \dot{V}_{\text{l,tr}}, \tag{79}$$

$$P_{\text{mus}} = \begin{cases} \frac{-P_{\text{mus,min}}}{T_{\text{I}} T_{\text{E}}} t^2 + \frac{P_{\text{mus,min}} T_{\text{R}}}{T_{\text{I}} T_{\text{E}}} t & \text{if } t \in [0, T_{\text{I}}], \\ \frac{P_{\text{mus,min}}}{1 - e^{-\frac{T_{\text{E}}}{\tau}}} \left(e^{-\frac{(t - T_{\text{I}})}{\tau}} - e^{-\frac{T_{\text{E}}}{\tau}} \right) & \text{if } t \in (T_{\text{I}}, T_{\text{R}}], \end{cases} \tag{80}$$

$$T_{\text{I}} + T_{\text{E}} = T_{\text{R}} = \frac{60}{RR}, \tag{81}$$

$$T_{\text{I}} = T_{\text{E}} \cdot \text{IE}_{\text{ratio}}, \tag{82}$$

$$\tau = \frac{T_{\text{E}}}{5}. \tag{83}$$

$$V_D = V_l + V_{tr} + V_b. \tag{84}$$

Gas exchange and transport

The gas exchange and transport model comprises the lung gas exchange, the tissue gas exchange and the gas transport through blood circulation. The equations are taken from [2].

O_2 and CO_2 dissociation curves.

The equations account for Haldane and Bohr effects, i.e. the dependence of hemoglobin affinity to each gas is also a function of the amount of the other gas present. Here, the model output variables are C which denotes the gas concentration (measured in $\text{mL}_{\text{gas}}/\text{mL}_{\text{blood}}$) and P which indicates the pressure (expressed in mmHg). The dissociation curves allow us to easily derive gas partial pressure from gas concentration and viceversa. The values of the parameters appearing in equations (85)-(96) are taken from [2] and are reported in Table 5.

Variables subscripts: 1, O_2; 2, CO_2.

$$C_1 = C_{\mathrm{sat},1}\frac{F_1^{\frac{1}{h_1}}}{1+F_1^{\frac{1}{h_1}}}, \tag{85}$$

$$C_2 = C_{\mathrm{sat},2}\frac{F_2^{\frac{1}{h_2}}}{1+F_2^{\frac{1}{h_2}}}, \tag{86}$$

$$F_1 = P_1\frac{1+\beta_1 P_2}{k_1(1+\alpha_1 P_2)}, \tag{87}$$

$$F_2 = P_2\frac{1+\beta_2 P_1}{k_2(1+\alpha_2 P_1)}. \tag{88}$$

$$P_1 = r_1 + (r_1^2 - s_1)^{1/2}, \tag{89}$$

$$P_2 = D_2\frac{(1+\alpha_2 P_1)}{(1+\beta_2 P_1)}, \tag{90}$$

$$r_1 = -(1+\beta_1 D_2 - \beta_2 D_1 - \alpha_1\alpha_2 D_1 D_2)/(2(\beta_2 + \alpha_2\beta_1 D_2)), \tag{91}$$

$$r_2 = -(1+\beta_2 D_1 - \beta_1 D_2 - \alpha_2\alpha_1 D_2 D_1)/(2(\beta_1 + \alpha_1\beta_2 D_1)), \tag{92}$$

$$s_1 = -(D_1 + \alpha_1 D_1 D_2)/(\beta_2 + \alpha_2\beta_1 D_2), \tag{93}$$

$$s_2 = -(D_2 + \alpha_2 D_2 D_1)/(\beta_1 + \alpha_1\beta_2 D_1), \tag{94}$$

$$D_1 = k_1\left[\frac{C_1}{C_{\mathrm{sat},1} - C_1}\right]^{h_1}, \tag{95}$$

$$D_2 = k_2\left[\frac{C_2}{C_{\mathrm{sat},2} - C_2}\right]^{h_2}. \tag{96}$$

Lung gas exchange

The lung gas exchange model is based on conservation of mass principles, i.e. gas balance for the dead space depends on flow between this space, the atmosphere and the alveoli, whereas gas balance for the alveoli depends on flow between this space, the dead space, as well as gas exchange with the pulmonary capillaries. Circulatory transport delays are also included to account for the time that it takes for blood to transport gases from the lungs to the systemic tissues τ_{LT} and from the thoracic veins back to the pulmonary capillaries τ_{VL}. The equations are taken from [2], with small modifications. In this section, the model variables are the following: F denotes the gas volume fraction (dimensionless), $\dot{V}$ denotes the airflow (measured in mL/s), V denotes the volume (expressed in mL), P is the pressure (measured in mmHg), C indicates the concentration of gas in blood, while $\tilde{C}$ denotes the concentrations of gas after the transport delay (both expressed in $\mathrm{mL_{gas}/mL_{blood}}$), and q is the blood flow (measured in mL/s). When possible the values of the parameters appearing in equations (98)-(111) are taken from [2], otherwise they are calibrated to match physiological model output indexes (see Table 9 for main model-predicted cardiovascular and respiratory indexes and reference values reported in the literature). The parameters in equations (97)-(110) are reported in Table 5.

Variables subscripts: D, dead space; A, alveoli; ao, airway opening; l, larinx; b, bronchea; pp, peripheral pulmonary arteries; ps, pulmonary shunt; v, thoracic veins; a, systemic arteries.

$$\frac{dF_{\mathrm{D,O_2}}}{dt} = \frac{1}{V_{\mathrm{D}}}\left[u(\dot{V}_{\mathrm{ao,l}})\dot{V}_{\mathrm{ao,l}}\left(F_{\mathrm{i,O_2}} - F_{\mathrm{D,O_2}}\right) + u(-\dot{V}_{\mathrm{b,A}})\dot{V}_{\mathrm{b,A}}\left(F_{\mathrm{D,O_2}} - F_{\mathrm{A,O_2}}\right)\right], \tag{97}$$

$$\frac{dF_{\mathrm{D,CO_2}}}{dt} = \frac{1}{V_{\mathrm{D}}}\left[u(\dot{V}_{\mathrm{ao,l}})\dot{V}_{\mathrm{ao,l}}\left(F_{\mathrm{i,CO_2}} - F_{\mathrm{D,CO_2}}\right) + u(-\dot{V}_{\mathrm{b,A}})\dot{V}_{\mathrm{b,A}}\left(F_{\mathrm{D,CO_2}} - F_{\mathrm{A,CO_2}}\right)\right], \tag{98}$$

$$\frac{dF_{\mathrm{A,O_2}}}{dt} = \frac{1}{V_{\mathrm{A}}}\left(u(\dot{V}_{\mathrm{ao,l}})\dot{V}_{\mathrm{b,A}}(F_{\mathrm{D,O_2}} - F_{\mathrm{A,O_2}}) - K\dot{M}_{\mathrm{O_2}}\right), \tag{99}$$

$$\frac{dF_{\mathrm{A,CO_2}}}{dt} = \frac{1}{V_{\mathrm{A}}}\left(u(\dot{V}_{\mathrm{ao,l}})\dot{V}_{\mathrm{b,A}}(F_{\mathrm{D,CO_2}} - F_{\mathrm{A,CO_2}}) - K\dot{M}_{\mathrm{CO_2}}\right), \tag{100}$$

where $u(\cdot)$ is the *Heaviside step function* and

$$\dot{M}_{\mathrm{O_2}} = K_{\mathrm{O_2}}(P_{\mathrm{A,O_2}} - P_{\mathrm{pp,O_2}})\,, \tag{101}$$

$$\dot{M}_{\mathrm{CO_2}} = K_{\mathrm{CO_2}}(P_{\mathrm{A,CO_2}} - P_{\mathrm{pp,CO_2}})\,. \tag{102}$$

$$\frac{dC_{\mathrm{pp,O_2}}}{dt} = \frac{1}{V_{\mathrm{pp}}}(q_{\mathrm{pp}}(\tilde{C}_{\mathrm{v,O_2}} - C_{\mathrm{pp,O_2}}) + \dot{M}_{\mathrm{O_2}})\,, \tag{103}$$

$$\frac{dC_{\mathrm{pp,CO_2}}}{dt} = \frac{1}{V_{\mathrm{pp}}}(q_{\mathrm{pp}}(\tilde{C}_{\mathrm{v,CO_2}} - C_{\mathrm{pp,CO_2}}) + \dot{M}_{\mathrm{CO_2}})\,. \tag{104}$$

$$P_{\mathrm{A,O_2}} = F_{\mathrm{A,O_2}} \cdot (P_{\mathrm{atm}} - P_{\mathrm{ws}}), \tag{105}$$

$$P_{\mathrm{A,CO_2}} = F_{\mathrm{A,CO_2}} \cdot (P_{\mathrm{atm}} - P_{\mathrm{ws}}). \tag{106}$$

$$C_{\mathrm{a,O_2}} = \max\left(\frac{q_{\mathrm{pp}}C_{\mathrm{pp,O_2}} + q_{\mathrm{ps}}\tilde{C}_{\mathrm{v,O_2}}}{q_{\mathrm{pp}} + q_{\mathrm{ps}}}, 0\right), \tag{107}$$

$$C_{\mathrm{a,CO_2}} = \max\left(\frac{q_{\mathrm{pp}}C_{\mathrm{pp,CO_2}} + q_{\mathrm{ps}}\tilde{C}_{\mathrm{v,CO_2}}}{q_{\mathrm{pp}} + q_{\mathrm{ps}}}, 0\right). \tag{108}$$

$$S_{\mathrm{a,O_2}}\% = \frac{C_{\mathrm{a,O_2}} - C_{\mathrm{a,O_2 diss}}}{1.34\,hgb}100, \tag{109}$$

$$C_{\mathrm{a,O_2 diss}} = \begin{cases} P_{\mathrm{a,O_2}}\frac{0.003}{100}, & \text{if } \left(C_{\mathrm{sat,O_2}} - 1.34\,hgb - P_{\mathrm{a,O_2}}\frac{0.003}{100}\right) \geq 0, \\ C_{\mathrm{sat,O_2}} - 1.34\,hgb, & \text{otherwise.} \end{cases} \tag{110}$$

Tissue gas exchange

The tissue gas exchange model accounts for the O_2 consumption and CO_2 production of tissues and organs at the level of the five systemic peripheral compartments (coronary, brain, skeletal muscle, splanchnic, and extrasplanchnic), whereas the venous pool gas transport model describes O_2 and CO_2 transport through the systemic and thoracic veins. Moreover, we assume that blood and tissue form an homogeneous mixture with total volume given by the (fixed) tissue volume and the (time-varying) blood volume. Here, the model variables are the following: C indicates the concentration of gas in blood, $\tilde{C}$ is the delayed concentration (both measured in $\mathrm{mL_{gas}/mL_{blood}}$), V denotes the blood volume (expressed in mL), and q is the blood flow (expressed in mL/s). The values of the parameters appearing in equations (111)-(116) are taken from [2] and are reported in Table 5.

Variables subscripts: $\mathrm{j} = \{\mathrm{s, e, m, b, h}\}$ stands for the compartments of peripheral systemic circulation; p, peripheral arteries; v, peripheral veins; a; systemic arteries; v; thoracic veins.

$$\frac{dC_{\mathrm{jp,O_2}}}{dt} = \frac{1}{V_{\mathrm{T,jp}} + V_{\mathrm{jp}}} \cdot \left[q_{\mathrm{jp,in}}(\tilde{C}_{\mathrm{a,O_2}} - C_{\mathrm{jp,O_2}}) - \dot{M}_{\mathrm{O_2,jp}}\right], \tag{111}$$

$$\frac{dC_{\mathrm{jp,CO_2}}}{dt} = \frac{1}{V_{\mathrm{T,jp}} + V_{\mathrm{jp}}} \cdot \left[q_{\mathrm{jp,in}}(\tilde{C}_{\mathrm{a,CO_2}} - C_{\mathrm{jp,CO_2}}) + \dot{M}_{\mathrm{CO_2,jp}}\right]. \tag{112}$$

$$\frac{dC_{\mathrm{jv,O_2}}}{dt} = \frac{1}{V_{\mathrm{jv}}}[q_{\mathrm{jp}}(C_{\mathrm{jp,O_2}} - C_{\mathrm{jv,O_2}})], \tag{113}$$

$$\frac{dC_{\mathrm{jv,CO_2}}}{dt} = \frac{1}{V_{\mathrm{jv}}}[q_{\mathrm{jp}}(C_{\mathrm{jp,CO_2}} - C_{\mathrm{jv,CO_2}})]. \tag{114}$$

$$\frac{dC_{\text{v},\text{O}_2}}{dt} = \frac{1}{V_{\text{tv}}}\left[\sum_j q_{\text{jv}}(C_{\text{jv},\text{O}_2} - C_{\text{v},\text{O}_2})\right], \tag{115}$$

$$\frac{dC_{\text{v},\text{CO}_2}}{dt} = \frac{1}{V_{\text{tv}}}\left[\sum_j q_{\text{jv}}(C_{\text{jv},\text{CO}_2} - C_{\text{v},\text{CO}_2})\right]. \tag{116}$$

Cardiovascular control models

The control mechanisms that are modeled here are autoregulation, autonomic nervous system (ANS) (baro-, chemo- and lung stretch receptors), and central nervous system (CNS) ischemic response. The autoregulation monitors the venous oxygen concentration and the venous carbon dioxide concentration by acting on the brain peripheral, the coronary peripheral and the muscle peripheral resistances. The nervous system mediated control is able to monitor the systemic arterial pressure P_{sa}, the arterial oxygen and carbon dioxide concentrations C_{a,CO_2} and C_{a,CO_2} and the tidal lung volume V_{td}. These signals affect fiber firing rates: afferent baroreflex pathway f_{ab}, afferent chemoreflex pathway f_{apc} and afferent pulmonary stretch receptor pathway f_{ap}. After that, the CNS processes the signals from the afferent pathways and generates a response to possible lack of O_2 by changing the firing rate of peripheral circulation sympathetic f_{sp}, heart sympathetic f_{sh}, venous circulation sympathetic f_{sv} and vagal para-sympathetic efferent pathways f_{v}. Then, efferent pathways stimulate changes of properties in vessels (peripheral resistances and unstressed volumes), changes of properties in cardiac chambers (maximum elastance), and heart period.

Local metabolic regulation

In this section, the equations are the ones proposed in [11]. The model variables are denotes as x which represent the effect of O_2 and CO_2 on cerebral, coronary or muscular circulation, R which indicates the resistances (measured in mmHg s/mL); C which is the gas concentration in the blood (expressed in $\text{mL}_{\text{gas}}/\text{mL}_{\text{blood}}$); P that represents the gas partial pressure (measured in mmHg). The parameters in equations (117)-(125) are taken from [11] and are reported in Table 6.

Variables subscripts: bp; brain peripheral arteries; hp, coronary peripheral arteries; mp, skeletal muscle peripheral arteries.

$$G_{\text{bp}} = 1 - g_{\text{b},\text{O}_2} \cdot x_{\text{b},\text{O}_2} + x_{\text{b},\text{CO}_2}, \tag{117}$$

$$\frac{dx_{\text{b},\text{O}_2}}{dt} = \frac{1}{\tau_{\text{O}_2}} \cdot \left[-x_{\text{b},\text{O}_2} + (C_{\text{bp},\text{O}_2} - C_{\text{vb},\text{O}_2 0})\right], \tag{118}$$

$$R_{\text{bp}} = \frac{R_{\text{bp0}}}{G_{\text{bp}}}, \tag{119}$$

$$\phi_{\text{b}}(P_{\text{a},\text{CO}_2}) = \frac{A + \frac{B}{1+CC\cdot e^{D\cdot\log(P_{\text{a},\text{CO}_2})}}}{A + \frac{B}{1+CC\cdot e^{D\cdot\log(P_{\text{a},\text{CO}_2 0})}}} - 1, \tag{120}$$

$$\frac{dx_{\text{b},\text{CO}_2}}{dt} = \frac{1}{\tau_{\text{CO}_2}} \cdot \left[-x_{\text{b},\text{CO}_2} + \phi_{\text{b}}(P_{\text{a},\text{CO}_2})\right]. \tag{121}$$

$$R_{\text{jp}} = R_{\text{jp0}} \cdot \frac{(1 + x_{\text{j},\text{CO}_2})}{(1 - g_{\text{j},\text{O}_2} \cdot x_{\text{j},\text{O}_2})}, \qquad \text{with j} = \{\text{h}, \text{m}\}, \tag{122}$$

$$\frac{dx_{\text{j},\text{O}_2}}{dt} = \frac{1}{\tau_{\text{O}_2}} \cdot \left[-x_{\text{j},\text{O}_2} + (C_{\text{jp},\text{O}_2} - C_{\text{vj},\text{O}_2 0})\right], \tag{123}$$

$$\phi_{\text{j}}(P_{\text{a},\text{CO}_2}) = \frac{1 - e^{\frac{P_{\text{a},\text{CO}_2} - P_{\text{a},\text{CO}_2 0}}{k_{\text{j},\text{CO}_2}}}}{1 + e^{\frac{P_{\text{a},\text{CO}_2} - P_{\text{a},\text{CO}_2 0}}{k_{\text{j},\text{CO}_2}}}}, \tag{124}$$

$$\frac{dx_{\text{j},\text{CO}_2}}{dt} = \frac{1}{\tau_{\text{CO}_2}} \cdot \left[-x_{\text{j},\text{CO}_2} + \phi_{\text{j}}(P_{\text{a},\text{CO}_2})\right]. \tag{125}$$

ANS afferent pathways

The ANS afferent pathways comprises the baroreflex, the chemoreflex and the lung-strech reflectors mechanisms. In this section, the model variables are the blood pressure P (measured in mmHg), the gas concentration C (measured in $\mathrm{mL_{gas}/mL_{blood}}$), the volume V (measured in mL) and the firing rate f (measured in spikes/s). In this section, the equations for baroreflex and lung-stretch receptors, as well as theirs parameters, are taken from [18], whereas the equations and the parameters for the chemoreflex are taken from [19]. Table 6 reports the parameters in equations (126)-(136).

Variables subscripts: sa, systemic arteries; ab, afferent baroreflex pathway; c,stat, chemoreceptor frequency discharge in static conditions; c,dyn, chemoreceptor frequency discharge in dynamic conditions; apc; afferent chemoreflex pathway; ap, afferent lung stretch receptor pathway; td, tidal lung space.

Afferent baroreflex pathway

$$\tau_{\mathrm{p,b}} \cdot \frac{d\tilde{P}}{dt} = P_{\mathrm{sa}} + \tau_{\mathrm{z,b}} \cdot \frac{dP_{\mathrm{sa}}}{dt} - \tilde{P}, \tag{126}$$

$$f_{\mathrm{ab}} = \frac{f_{\mathrm{ab,min}} + f_{\mathrm{ab,max}} \cdot e^{\frac{\tilde{P}-P_{\mathrm{n}}}{k_{\mathrm{ab}}}}}{1 + e^{\frac{\tilde{P}-P_{\mathrm{n}}}{k_{\mathrm{ab}}}}}. \tag{127}$$

Afferent chemoreflex pathway

$$X_{\mathrm{O_2}} = AA\,(1 - S_{\mathrm{a,O_2}}) + BB, \tag{128}$$

$$\Phi_{\mathrm{stat}} = K_{\mathrm{CO_2}}\,(C_{\mathrm{a,CO_2}} - C_{\mathrm{a,CO_2 0}}) \cdot K_{\mathrm{O_2}} \left(1 - e^{-X_{\mathrm{O_2}}/K_{\mathrm{O_2}}}\right), \tag{129}$$

$$f_{\mathrm{c,stat}} = \begin{cases} K_{\mathrm{stat}}\left(1 - e^{-\Phi_{\mathrm{stat}}/K_{\mathrm{stat}}}\right) & \text{if } C_{\mathrm{a,CO_2}} \geqslant C_{\mathrm{a,CO_2 0}} \\ 0 & \text{if } C_{\mathrm{a,CO_2}} < C_{\mathrm{a,CO_2 0}} \end{cases}, \tag{130}$$

$$\frac{d\varphi_{\mathrm{CO_2,dyn}}}{dt} = \frac{1}{\tau_{\mathrm{cap}}}\left(\tau_{\mathrm{cc,CO_2 dyn}}\frac{dC_{\mathrm{a,CO_2}}}{dt} - \varphi_{\mathrm{CO_2,dyn}}\right), \tag{131}$$

$$f_{\mathrm{c,dyn}} = K_{\mathrm{dyn}}(1 - e^{-\varphi_{\mathrm{CO_2,dyn}}/K_{\mathrm{dyn}}}), \tag{132}$$

$$\frac{d\varphi_{\mathrm{apc}}}{dt} = \frac{1}{\tau_{\mathrm{cap}}}\left(f_{\mathrm{c,stat}} + f_{\mathrm{c,dyn}} - \varphi_{\mathrm{apc}}\right), \tag{133}$$

$$f_{\mathrm{apc}} = \max\left(\varphi_{\mathrm{apc}}, 0\right). \tag{134}$$

Afferent activity from lung stretch receptors

$$\varphi_{\mathrm{ap}}(V_{\mathrm{td}}) = G_{\mathrm{ap}} \cdot V_{\mathrm{td}}, \tag{135}$$

$$\frac{df_{\mathrm{ap}}}{dt} = \frac{1}{\tau_{\mathrm{p}}} \cdot (-f_{\mathrm{ap}} + \varphi_{\mathrm{ap}}). \tag{136}$$

CNS ischemic response

The model variables for this section are $\Delta\theta_{\mathrm{O_2,sj}}$ that represents the change in the offset term caused by the CNS hypoxia, and $\Delta\theta_{\mathrm{CO_2,sj}}$ which represents the change in the offset term caused by central chemoreceptor stimulation. The equations (137)-(140) are taken from [11], as well as for theirs parameters, which are reported in Table 6.

Variables subscripts: sp, efferent peripheral circulation sympathetic pathway; sh, efferent heart

sympathetic pathway; sv, efferent venous circulation sympathetic pathway.

$$\omega_{\mathrm{sj}}(P_{\mathrm{a,O_2}}) = \frac{\chi_{\mathrm{sj}}}{1+e^{\frac{P_{\mathrm{a,O_2}}-\tilde{P}_{\mathrm{O_2,sj}}}{k_{\mathrm{isc,sj}}}}}, \quad \text{with } \mathrm{j}=\{\mathrm{p,v,h}\}, \tag{137}$$

$$\frac{d\Delta\theta_{\mathrm{O_2,sj}}}{dt} = \frac{1}{\tau_{\mathrm{isc}}}\cdot(-\Delta\theta_{\mathrm{O_2,sj}}+\omega_{\mathrm{sj}}), \tag{138}$$

$$\frac{d\Delta\theta_{\mathrm{CO_2,sj}}}{dt} = \frac{1}{\tau_{\mathrm{cc}}}\cdot[-\Delta\theta_{\mathrm{CO_2,sj}}+(P_{\mathrm{a,CO_2}}-P_{\mathrm{a,CO_20}})], \tag{139}$$

$$\theta_{\mathrm{sj}} = \theta_{\mathrm{sjn}} - \Delta\theta_{\mathrm{O_2,sj}} - g_{\mathrm{ccsj}}\cdot\Delta\theta_{\mathrm{CO_2,sj}}. \tag{140}$$

ANS efferent pathways

The model variables for this section are the sympathetic fiber firing rates denoted by f. The equations and the parameters for the efferent sympathetic pathway are taken from [11], whereas the ones for the efferent vagal pathway are taken from [18]. Some parameters are modified from the values reported in [11, 18] in order to match physiological model output indexes (see Table 9 for main model-predicted cardiovascular and respiratory indexes and reference values reported in the literature). The parameters in equations (141)-(142) are reported in Table 7.

Variables subscripts: sp, efferent peripheral circulation sympathetic pathway; sh, efferent heart sympathetic pathway; sv, efferent venous circulation sympathetic pathway; ab, afferent baroreflex pathway; apc, afferent chemoreflex pathway; ap, afferent lung stretch receptor pathway; v, efferent vagal para-sympathetic pathway.

Efferent sympathetic pathway

$$f_{\mathrm{sj}} = \begin{cases} f_{\mathrm{es,min}}, & \text{if } f_{\mathrm{sj}} \le f_{\mathrm{es,min}}, \\ f_{\mathrm{es,\infty}} + (f_{\mathrm{es,0}} - f_{\mathrm{es,\infty}})\cdot e^{k_{\mathrm{es}}\cdot\left[W_{\mathrm{b,sj}}\cdot f_{\mathrm{ab}} + W_{\mathrm{c,sj}}\cdot f_{\mathrm{apc}} + W_{\mathrm{p,sj}}\cdot f_{\mathrm{ap}} - \theta_{\mathrm{sj}}\right]}, & \text{if } f_{\mathrm{es,min}} < f_{\mathrm{sj}} < f_{\mathrm{es,max}}, \\ f_{\mathrm{es,max}}, & \text{if } f_{\mathrm{sj}} \ge f_{\mathrm{es,max}}, \end{cases}$$
$$\text{with } \mathrm{j}=\{\mathrm{h,p,v}\}. \tag{141}$$

Efferent vagal pathway

$$f_{\mathrm{v}} = \frac{f_{\mathrm{ev,0}} + f_{\mathrm{ev,\infty}}\cdot e^{\frac{f_{\mathrm{ab}}-f_{\mathrm{ab,0}}}{k_{\mathrm{ev}}}}}{1+e^{\frac{f_{\mathrm{ab}}-f_{\mathrm{ab,0}}}{k_{\mathrm{ev}}}}} + W_{\mathrm{c,v}}\cdot f_{\mathrm{apc}} + W_{\mathrm{p,v}}\cdot f_{\mathrm{ap}} - \theta_{\mathrm{v}} \tag{142}$$

Effectors for reflex regulation

In this section, the model variables are the output of the static characteristic θ, the efferent firing rates f, the time t, the changes in heart period induced by sympathetic stimulation ΔT_{s} and the changes in heart period induced by vagal stimulation ΔT_{v}. The equations and the parameters are taken from [18]. The parameters in equations (143)-(157) are reported in Table 7.

Variables subscripts: sp, splanchnic peripheral arteries; ep, extra-splanchnic peripheral arteries; mp, skeletal muscle peripheral arteries; sv, splanchnic peripheral veins; ev, extra-splanchnic peripheral veins; mv, skeletal muscle peripheral veins; sp, efferent peripheral circulation sympathetic pathway; sh, efferent heart sympathetic pathway; sv, efferent venous circulation sympathetic pathway.

$$\sigma_\theta = \begin{cases} G_\theta\cdot\ln(f_{\mathrm{sp}}(t-D_\theta) - f_{\mathrm{es,min}}+1), & \text{if } f_{\mathrm{sp}} \ge f_{\mathrm{es,min}}, \\ 0, & \text{if } f_{\mathrm{sp}} < f_{\mathrm{es,min}} \end{cases}, \quad \text{with } \theta=\{R_{\mathrm{mp}}, R_{\mathrm{sp}}, R_{\mathrm{ep}}\}, \tag{143}$$

$$\frac{d\Delta\theta}{dt} = \frac{1}{\tau_\theta}\cdot(-\Delta\theta+\sigma_\theta), \tag{144}$$

$$\theta(t) = \Delta\theta(t) + \theta_0. \tag{145}$$

$$\tag{146}$$

$$\sigma_\theta = \begin{cases} G_\theta \cdot \ln(f_{\rm sv}(t-D_\theta) - f_{\rm es,min} + 1), & \text{if } f_{\rm sp} \geq f_{\rm es,min}, \\ 0, & \text{if } f_{\rm sp} < f_{\rm es,min}, \end{cases}, \qquad \text{with } \theta = \{V_{\rm u,mv}, V_{\rm u,sv}, V_{\rm u,ev}\}, \tag{147}$$

$$\frac{d\Delta\theta}{dt} = \frac{1}{\tau_\theta} \cdot (-\Delta\theta + \sigma_\theta), \tag{148}$$

$$\theta(t) = \Delta\theta(t) + \theta_0. \tag{149}$$

$$\sigma_\theta = \begin{cases} G_\theta \cdot \ln(f_{\rm sh}(t-D_\theta) - f_{\rm es,min} + 1) & \text{if } f_{\rm sh} \geq f_{\rm es,min} \\ 0 & \text{if } f_{\rm sh} < f_{\rm es,min} \end{cases}, \qquad \text{with } \theta = \{E_{\rm max,LV}, E_{\rm max,RV}\}, \tag{150}$$

$$\frac{d\Delta\theta}{dt} = \frac{1}{\tau_\theta} \cdot (-\Delta\theta + \sigma_\theta), \tag{151}$$

$$\theta(t) = \Delta\theta(t) + \theta_0. \tag{152}$$

$$\sigma_{\rm T,s}(t) = \begin{cases} G_{\rm T,s} \cdot \ln\left(f_{\rm sh}(t-D_{\rm T,s}) - f_{\rm es,min} + 1\right), & \text{if } f_{\rm sh} \geq f_{\rm es,min} \\ 0, & \text{if } f_{\rm sh} < f_{\rm es,min}, \end{cases} \tag{153}$$

$$\frac{d\Delta T_{\rm s}(t)}{dt} = \frac{1}{\tau_{\rm T,s}} \cdot (-\Delta T_{\rm s}(t) + \sigma_{\rm T,s}(t)), \tag{154}$$

$$\sigma_{\rm T,v}(t) = G_{\rm T_v} \cdot f_{\rm v}(t - D_{\rm T_v}), \tag{155}$$

$$\frac{d\Delta T_{\rm v}(t)}{dt} = \frac{1}{\tau_{\rm T_v}} \cdot (-\Delta T_{\rm v}(t) + \sigma_{\rm T,v}(t)), \tag{156}$$

$$T = \Delta T_{\rm s} + \Delta T_{\rm v} + T_0. \tag{157}$$

Respiratory control models

The respiratory control mechanisms is able to change the amplitude of respiratory muscle pressure and the respiratory period, by monitoring $P_{\rm a,CO_2}$ by means of central chemoreceptors, and $P_{\rm a,O_2}$ and $P_{\rm a,CO_2}$ by to peripheral chemoreceptors. The model variables are the respiratory rate RR and the $P_{\rm mus}$ amplitude $P_{\rm mus,min}$. The equations and the parameters are taken from [2] with some modifications on the parameters in order to match physiological model output indexes (see Table 9 for main model-predicted cardiovascular and respiratory indexes and reference values reported in the literature). The parameters in equations (158)-(165) are reported in Table 8.

Variables subscripts: c, central chemoreceptors; p, peripheral chemoreceptors; mus; respiratory muscles.

$$P_{\rm mus,min} = -\max\left(0, -P_{\rm mus,min0} + \left(\frac{\Delta P_{\rm mus,min_c} + \Delta P_{\rm mus,min_p}}{980}\right)\right), \tag{158}$$

$$RR = RR_0 + \Delta RR_{\rm c} + \Delta RR_{\rm p}. \tag{159}$$

$$\frac{d\Delta P_{\rm mus,min_c}}{dt} = \frac{-\Delta P_{\rm mus,min_c} + G_{\rm c,A} \cdot u_{\rm c}}{\tau_{\rm c,A}}, \tag{160}$$

$$\frac{d\Delta RR_{\rm c}}{dt} = \frac{-\Delta RR_{\rm c} + G_{\rm c,f} \cdot u_{\rm c}}{\tau_{\rm c,f}}, \tag{161}$$

$$u_{\rm c}(t) = [P_{\rm a,CO_2} - P_{\rm a,CO_2 0}](t - D_{\rm c}). \tag{162}$$

$$\frac{d\Delta P_{\text{mus,min}_\text{p}}}{dt} = \frac{-\Delta P_{\text{mus,min}_\text{p}} + G_{\text{p,A}} \cdot u_\text{p}}{\tau_{\text{p,A}}}, \tag{163}$$

$$\frac{d\Delta RR_\text{p}}{dt} = \frac{-\Delta RR_\text{p} + G_{\text{p,f}} \cdot u_\text{p}}{\tau_{\text{p,f}}}, \tag{164}$$

$$u_\text{p}(t) = [f_{\text{apc}} - f_{\text{apc},0}](t - D_\text{p}). \tag{165}$$

Table 1: **Baseline parameters in equations (1)-(36).**

**Parameter**	**Notation**	**Value**	**Unit**
**Systemic arteries**			
Systemic arterial compliance	C_{sa}	0.20	$\text{mL} \cdot \text{mmHg}^{-1}$
Systemic arterial unstressed volume	$V_{\text{u,sa}}$	0	mL
Inlet peripheral resistance	R_{p}	0.001	$\text{mmHg} \cdot \text{s} \cdot \text{mL}^{-1}$
**Systemic peripheral arteries**			
Splanchnic peripheral compliance	C_{sp}	0.69	$\text{mL} \cdot \text{mmHg}^{-1}$
Splanchnic peripheral unstressed volume	$V_{\text{u,sp}}$	274.40	mL
Extra-splanchnic peripheral compliance	C_{ep}	0.64	$\text{mL} \cdot \text{mmHg}^{-1}$
Extra-splanchnic peripheral unstressed volume	$V_{\text{u,ep}}$	134.64	mL
Skeletal muscle peripheral compliance	C_{mp}	0.57	$\text{mL} \cdot \text{mmHg}^{-1}$
Skeletal muscle peripheral unstressed volume	$V_{\text{u,mp}}$	105.80	mL
Brain peripheral compliance	C_{bp}	0.36	$\text{mL} \cdot \text{mmHg}^{-1}$
Brain peripheral unstressed volume	$V_{\text{u,bp}}$	72.13	mL
Coronary peripheral compliance	C_{hp}	0.10	$\text{mL} \cdot \text{mmHg}^{-1}$
Coronary peripheral unstressed volume	$V_{\text{u,hp}}$	24.00	mL
**Systemic veins**			
Splanchnic venous compliance	C_{sv}	42.78	$\text{mL} \cdot \text{mmHg}^{-1}$
Splanchnic venous resistance	R_{sv}	0.04	$\text{mmHg} \cdot \text{s} \cdot \text{mL}^{-1}$
Extra-splanchnic venous compliance	C_{ev}	14.00	$\text{mL} \cdot \text{mmHg}^{-1}$
Extra-splanchnic venous resistance	R_{ev}	0.04	$\text{mmHg} \cdot \text{s} \cdot \text{mL}^{-1}$
Skeletal muscle venous compliance	C_{mv}	11.00	$\text{mL} \cdot \text{mmHg}^{-1}$
Skeletal muscle venous resistance	R_{mv}	0.05	$\text{mmHg} \cdot \text{s} \cdot \text{mL}^{-1}$
Brain venous compliance	C_{bv}	7.50	$\text{mL} \cdot \text{mmHg}^{-1}$
Brain venous unstressed volume	$V_{\text{u,bv}}$	294.64	mL
Brain venous resistance	R_{bv}	0.08	$\text{mmHg} \cdot \text{s} \cdot \text{mL}^{-1}$
Coronary venous compliance	C_{hv}	2.50	$\text{mL} \cdot \text{mmHg}^{-1}$
Coronary venous unstressed volume	$V_{\text{u,hv}}$	98.21	mL
Coronary venous resistance	R_{hv}	0.22	$\text{mmHg} \cdot \text{s} \cdot \text{mL}^{-1}$
Thoracic vein pressure-volume parameter	D_1	0.39	mmHg
Thoracic vein pressure-volume parameter	D_2	-5.00	mmHg
Thoracic vein pressure-volume parameter	K_1	0.15	$\text{mmHg} \cdot \text{mL}^{-1}$
Thoracic vein pressure-volume parameter	K_2	0.40	mmHg
Thoracic vein pressure-volume parameter	K_{xp}	2.00	mmHg
Thoracic vein pressure-volume parameter	K_{xv}	8.00	mmHg
Thoracic vein unstressed volume	$V_{\text{u,tv}}$	130.00	mL
Thoracic vein minimum volume	$V_{\text{tv,min}}$	50.00	mL
Thoracic vein maximum volume	$V_{\text{tv,max}}$	350.00	mL
Thoracic vein resistance parameter	K_{R}	0.001	$\text{mmHg} \cdot \text{s} \cdot \text{mL}^{-1}$
Thoracic vein basal resistance	R_{tv0}	0.03	$\text{mmHg} \cdot \text{s} \cdot \text{mL}^{-1}$
**Pulmonary arteries**			
Pulmonary inlet peripheral resistance	$R_{\text{pp,in}}$	0.0001	$\text{mmHg} \cdot \text{s} \cdot \text{mL}^{-1}$
Shunt inlet peripheral resistance	$R_{\text{ps,in}}$	0.10	$\text{mmHg} \cdot \text{s} \cdot \text{mL}^{-1}$
Pulmonary artery unstressed volume	$V_{\text{u,pa}}$	0.00	mL
Pulmonary artery compliance	C_{pa}	0.76	$\text{mL} \cdot \text{mmHg}^{-1}$
**Pulmonary peripheral arteries**			
Pulmonary peripheral resistance	R_{pp}	0.0894	$\text{mmHg} \cdot \text{s} \cdot \text{mL}^{-1}$
Pulmonary peripheral unstressed volume	$V_{\text{u,pp}}$	108.24	mL
Pulmonary peripheral arteries compliance	C_{pp}	5.80	$\text{mL} \cdot \text{mmHg}^{-1}$
Pulmonary shunt	sh	1.70	%
**Pulmonary peripheral veins**			
Pulmonary veins resistance	R_{pv}	0.0056	$\text{mmHg} \cdot \text{s} \cdot \text{mL}^{-1}$
Pulmonary veins unstressed volume	$V_{\text{u,pv}}$	105.60	mL
Pulmonary veins compliance	C_{pv}	25.37	$\text{mL} \cdot \text{mmHg}^{-1}$

Table 2: **Baseline parameters in equations (37)-(52).**

Parameter	Notation	Value				Unit
**Cardiac valves**		**TV**	**PV**	**MV**	**AV**	
Effective length of the valve region	l^{eff}	1.00	1.50	1.00	1.00	cm
Parameter for valve regurgitation	M_{rg} $(\cdot 10^{-5})$	1.00	1.00	1.00	1.00	-
Parameter for valve stenosis	M_{st}	1.00	1.00	1.00	1.00	-
Area of the valve's annulus	A_{ann}	4.96	5.18	8.60	4.05	cm^2
Speed of valve opening	K_{open}	40.00	26.70	26.00	15.00	$\text{mmHg}^{-1} \cdot s^{-1}$
Speed of valve closing	K_{close}	53.30	26.70	53.30	15.00	$\text{mmHg}^{-1} \cdot \text{s}^{-1}$
Threshold value at which a valve opens	ΔP_{open}	0.00	0.00	0.00	0.00	mmHg
Threshold value at which a valve closes	ΔP_{close}	0.00	0.00	0.00	0.00	mmHg
Blood density	ρ	1.04				$\text{g} \cdot \text{mL}^{-1}$
Proximal systemic resistance	$R^{\text{S}}_{\text{prox}}$	0.005				$\text{mmHg} \cdot \text{s mL}^{-1}$
Proximal pulmonary resistance	$R^{\text{P}}_{\text{prox}}$	0.001				$\text{mmHg} \cdot \text{s mL}^{-1}$

Table 3: **Baseline parameters in equations (53)-(65).**

Parameter	Notation	Value		Unit
**Atria**		**LA**	**RA**	
Contraction time	$t_{\text{r}}T$	0.97	0.97	-
Relaxation time	$t_{\text{c}}T$	0.80	0.80	-
Relaxation period	T_{rp}/T	0.17	0.17	-
Contraction period	T_{cp}/T	0.17	0.17	-
Active elastance	E^{A}	0.07	0.06	$\text{mmHg} \cdot \text{mL}^{-1}$
Passive elastance	E^{B}	0.05	0.04	$\text{mmHg} \cdot \text{mL}^{-1}$
Viscoelastic coefficient of the cardiac wall	S $(\cdot 10^4)$	5.00	5.00	$\text{mmHg} \cdot \text{mL}^{-1}$
Unstressed volume	V_{u}	22.00	22.00	mL
**Ventricles**		**LV**	**RV**	
Constant for resistance	k_{R}	$3.75 \cdot 10^{-4}$	$1.4 \cdot 10^{-3}$	$\text{s} \cdot \text{mL}^{-1}$
Pressure parameter for pressure-volume curve	P_0	1.50	1.50	mmHg
Volume parameter for pressure-volume curve	k_{E}	0.014	0.011	mL^{-1}
Unstressed volume	V_{u}	14.76	35.90	mL
Parameter for systole duration	$T_{\text{sys},0}$	0.50		s
Parameter for systole duration	k_{sys}	0.075		s^2

Table 4: **Baseline parameters in equations (66)-(84).**

Parameter	Notation	Value	Unit
**Larynx, trachea, bronchi, alveoli**			
Larynx compliance	C_{l}	0.0013	$\text{L} \cdot \text{cmH}_2\text{O}^{-1}$
Larynx unstressed volume	$V_{\text{u,l}}$	34.40	mL
Resistance between mouth and larynx	$R_{\text{m,l}}$	1.02	$\text{cmH}_2\text{O} \cdot \text{s} \cdot \text{L}^{-1}$
Trachea compliance	C_{tr}	0.0024	$\text{L} \cdot \text{cmH}_2\text{O}^{-1}$
Trachea unstressed volume	$V_{\text{u,t}}$	6.63	mL
Resistance between larynx and trachea	$R_{\text{l,tr}}$	0.34	$\text{cmH}_2\text{O} \cdot \text{s} \cdot \text{L}^{-1}$
Bronchi compliance	C_{b}	0.013	$\text{L} \cdot \text{cmH}_2\text{O}^{-1}$
Bronchi unstressed volume	$V_{\text{u,b}}$	18.70	mL
Resistance between trachea and bronchi	$R_{\text{tr,b}}$	0.31	$\text{cmH}_2\text{O} \cdot \text{s} \cdot \text{L}^{-1}$
Alveoli compliance	C_{A}	0.20	$\text{L} \cdot \text{cmH}_2\text{O}^{-1}$
Alveoli unstressed volume	$V_{\text{u,A}}$	1.26	L
Resistance between bronchi and alveoli	$R_{\text{b,A}}$	0.08	$\text{cmH}_2\text{O} \cdot \text{s} \cdot \text{L}^{-1}$
**Pleural space**			
Chest wall compliance	C_{cw}	0.24	$\text{L} \cdot \text{cmH}_2\text{O}^{-1}$
Pleural pressure at end-expiration	$P_{\text{pl,EE}}$	-5.00	cmH_2O
Inspiratory-expiratory time ratio	IE_{ratio}	0.60	-

Table 5: **Baseline parameters in equations (85)-(116).**

**Parameter**	**Notation**	**Value**	**Unit**
**Dissociation Curves**			
Oxygen saturation capacity	$C_{\mathrm{sat,O_2}}$	0.2045	$\mathrm{mL}_{O_2} \cdot \mathrm{mL}_{\mathrm{blood}}^{-1}$
Carbon dioxide saturation capacity	$C_{\mathrm{sat,CO_2}}$	1.9564	$\mathrm{mL}_{O_2} \cdot \mathrm{mL}_{\mathrm{blood}}^{-1}$
Hill coefficient for oxygen	h_1	0.3836	-
Hill coefficient for carbon dioxide	h_2	1.8190	-
Oxygen dissociation slope	α_1	0.0320	mmHg^{-1}
Carbon dioxide dissociation slope	α_2	0.0559	mmHg^{-1}
Oxygen dissociation intercept	β_1	0.0083	mmHg^{-1}
Carbon dioxide dissociation intercept	β_2	0.0326	mmHg^{-1}
Oxygen dissociation constant	k_1	14.99	mmHg
Carbon dioxide dissociation constant	k_2	194.40	mmHg
**Lung gas exchange**			
Fraction of inspired oxygen	$F_{\mathrm{i,O_2}}$	21.04	%
Fraction of inspired carbon dioxide	$F_{\mathrm{i,CO_2}}$	0.0421	%
Constant for conversion of volumes	K	1.2103	-
Oxygen exchange coefficient	$K_{\mathrm{O_2}}$	2.28	$\mathrm{mL}_{O_2} \cdot (\mathrm{mL}_{\mathrm{blood}} \cdot \mathrm{s} \cdot \mathrm{mmHg})^{-1}$
Carbon dioxide exchange coefficient	$K_{\mathrm{CO_2}}$	65.60	$\mathrm{mL}_{CO_2} \cdot (\mathrm{mL}_{\mathrm{blood}} \cdot \mathrm{s} \cdot \mathrm{mmHg})^{-1}$
Atmospheric pressure	P_{atm}	760.00	mmHg
Water vapor pressure	P_{ws}	47.00	mmHg
Hemoglobin concentration	hgb	0.15	$\mathrm{g} \cdot \mathrm{mL}^{-1}$
**Tissue gas exchange**			
Splanchnic tissue volume	$V_{\mathrm{T,sp}}$	2673.00	mL
O_2 consumption rate in splanchnic	$\dot{M}_{\mathrm{O_2,sp}}$	108.4190	$\mathrm{mL} \cdot \mathrm{min}^{-1}$
CO_2 production rate in splanchnic	$\dot{M}_{\mathrm{CO_2,sp}}$	91.0720	$\mathrm{mL} \cdot \mathrm{min}^{-1}$
Extrasplanchnic tissue volume	$V_{\mathrm{T,ep}}$	262.00	mL
O_2 consumption rate in extrasplanchnic	$\dot{M}_{\mathrm{O_2,ep}}$	14.6830	$\mathrm{mL} \cdot \mathrm{min}^{-1}$
CO_2 production rate in extrasplanchnic	$\dot{M}_{\mathrm{CO_2,ep}}$	12.3337	$\mathrm{mL} \cdot \mathrm{min}^{-1}$
Skeletal muscles tissue volume	$V_{\mathrm{T,mp}}$	31200.00	mL
O_2 consumption rate in muscles	$\dot{M}_{\mathrm{O_2,mp}}$	51.60	$\mathrm{mL} \cdot \mathrm{min}^{-1}$
CO_2 production rate in muscles	$\dot{M}_{\mathrm{CO_2,mp}}$	43.3440	$\mathrm{mL} \cdot \mathrm{min}^{-1}$
Brain tissue volume	$V_{\mathrm{T,bp}}$	1300.00	mL
O_2 consumption rate in brain	$\dot{M}_{\mathrm{O_2,bp}}$	47.5020	$\mathrm{mL} \cdot \mathrm{min}^{-1}$
CO_2 production rate in brain	$\dot{M}_{\mathrm{CO_2,bp}}$	39.9017	$\mathrm{mL} \cdot \mathrm{min}^{-1}$
Coronaries tissue volume	$V_{\mathrm{T,hp}}$	284.00	mL
O_2 consumption rate in coronaries	$\dot{M}_{\mathrm{O_2,hp}}$	24.00	$\mathrm{mL} \cdot \mathrm{min}^{-1}$
CO_2 production rate in coronaries	$\dot{M}_{\mathrm{CO_2,hp}}$	20.16	$\mathrm{mL} \cdot \mathrm{min}^{-1}$
**Blood Transport Delays**			
Lungs to systemic tissues transport delay	τ_{LT}	18.00	s
Thoracic veins to lungs transport delay	τ_{VL}	10.00	s

Table 6: **Baseline parameters in equations (117)-(140).**

Parameter	Notation	Value	Unit
**Local metabolic regulation**			
Basal brain peripheral resistance	R_{bp0}	6.67	mmHg · s · mL^{-1}
Brain oxygen gain factor	$g_{\mathrm{b,O_2}}$	10.00	mL · mL^{-1}
Basal O_2 concentration in venous blood leaving the brain	$C_{\mathrm{vb,O_20}}$	0.14	-
Parameter for brain CO_2 autoregulation	A	20.90	-
Parameter for brain CO_2 autoregulation	B	92.80	-
Parameter for brain CO_2 autoregulation	CC	10570.00	-
Parameter for brain CO_2 autoregulation	D	-5.2510	-
Basal coronary peripheral resistance	R_{hp0}	19.71	mmHg · s · mL^{-1}
Heart oxygen gain factor	$g_{\mathrm{h,O_2}}$	35.00	mL · mL^{-1}
Basal O_2 concentration in venous blood leaving the heart	$C_{\mathrm{vh,O_20}}$	0.11	-
Parameter for heart CO_2 autoregulation	$k_{\mathrm{h,CO_2}}$	11.11	mmHg
Basal skeletal muscle peripheral resistance	R_{mp0}	2.11	mmHg · s · mL^{-1}
Muscle oxygen gain factor	$g_{\mathrm{m,O_2}}$	30.00	mL · mL^{-1}
Basal O_2 concentration in venous blood leaving the muscles	$C_{\mathrm{vm,O_20}}$	0.1550	-
Parameter for muscle CO_2 autoregulation	$k_{\mathrm{m,CO_2}}$	142.80	mmHg
O_2 time constant	$\tau_{\mathrm{O_2}}$	10.00	s
CO_2 time constant	$\tau_{\mathrm{CO_2}}$	20.00	s
**Afferent baroreflex pathways**			
Baroreflex time constant	$\tau_{\mathrm{z,b}}$	6.37	s
Baroreflex time constant	$\tau_{\mathrm{p,b}}$	2.08	s
Baroreflex minimum firing rate	$f_{\mathrm{ab,min}}$	2.52	spikes · s^{-1}
Baroreflex maximum firing rate	$f_{\mathrm{ab,max}}$	47.78	spikes · s^{-1}
Parameter for baroreflex firing rate	k_{ab}	11.76	mmHg
Baroreflex activation level	P_{n}	92.00	mmHg
**Afferent chemoreflex pathways**			
Parameter for chemoreflex response	AA	600.00	-
Parameter for chemoreflex response	BB	10.18	-
Basal arterial CO_2 concentration	$C_{\mathrm{a,CO_20}}$	0.36	-
Parameter for chemoreflex response	$K_{\mathrm{O_2}}$	200.00	-
Parameter for chemoreflex response	$K_{\mathrm{CO_2}}$	1.00	s^{-1}
Parameter for static chemoreflex response	K_{stat}	20.00	s^{-1}
Parameter for dynamic chemoreflex response	K_{dyn}	45.00	s^{-1}
Chemoreflex time constant	τ_{cap}	3.50	s
Chemoreflex time constant	$\tau_{\mathrm{cc,CO_2dyn}}$	600.00	s
**Afferent lung-stretch receptors pathways**			
Lung stretch receptors time constant	τ_{p}	2.00	s
Lung stretch receptors gain factor	G_{ap}	12.00	spikes · l^{-1} · s^{-1}
**CNS ischemic response**			
Saturation of peripheral hypoxic response	χ_{sp}	6.00	s^{-1}
Peripheral hypoxic response activation level	$\tilde{P}_{\mathrm{O_2sp}}$	30.00	mmHg
Parameter for peripheral hypoxic response	$k_{\mathrm{isc,sp}}$	2.00	mmHg
Saturation of venous hypoxic response	χ_{sv}	6.00	s^{-1}
Venous hypoxic response activation level	$\tilde{P}_{\mathrm{O_2sv}}$	30.00	mmHg
Parameter for venous hypoxic response	$k_{\mathrm{isc,sv}}$	2.00	mmHg
Saturation of heart hypoxic response	χ_{sh}	53.00	s^{-1}
Venous hypoxic response activation level	$\tilde{P}_{\mathrm{O_2sh}}$	45.00	mmHg
Parameter for heart hypoxic response	$k_{\mathrm{isc,sh}}$	6.00	mmHg
Hypoxic response time constant	τ_{isc}	30.00	s
Hypoxic response time constant	τ_{cc}	20.00	s
Basal arterial CO_2 concentration	$P_{\mathrm{a,CO_20}}$	40.00	mmHg
Peripheral offset term in basal condition	θ_{spn}	13.32	s^{-1}
Venous offset term in basal condition	θ_{svn}	13.32	s^{-1}
Heart offset term in basal condition	θ_{shn}	3.60	s^{-1}
Peripheral gain factor	$g_{\mathrm{cc,sp}}$	1.50	mmHg^{-1} · s^{-1}
Venous gain factor	$g_{\mathrm{cc,sv}}$	0.00	mmHg^{-1} · s^{-1}
Heart gain factor	$g_{\mathrm{cc,sh}}$	1.00	mmHg^{-1} · s^{-1}

Table 7: **Baseline parameters in equations (141)-(157).**

**Parameter**	**Notation**	**Value**	**Unit**
**Efferent sympathetic pathway**			
Baroreflex peripheral synaptic weight factor	$W_{\mathrm{b,sp}}$	-1.1375	-
Chemoreflex peripheral synaptic weight factor	$W_{\mathrm{c,sp}}$	1.56	-
Lung-stretch receptors peripheral synaptic weight factor	$W_{\mathrm{p,sp}}$	-0.4250	-
Baroreflex venous synaptic weight factor	$W_{\mathrm{b,sv}}$	-1.1375	-
Chemoreflex venous synaptic weight factor	$W_{\mathrm{c,sv}}$	1.56	-
Lung-stretch receptors venous synaptic weight factor	$W_{\mathrm{p,sv}}$	-0.4250	-
Baroreflex heart synaptic weight factor	$W_{\mathrm{b,sh}}$	-1.75	-
Chemoreflex heart synaptic weight factor	$W_{\mathrm{c,sh}}$	1.00	-
Lung-stretch receptors heart synaptic weight factor	$W_{\mathrm{p,sh}}$	0.00	-
Efferent sympathetic minimum firing rate	$f_{\mathrm{es,min}}$	2.66	spikes $\cdot$ s^{-1}
Efferent sympathetic maximum firing rate	$f_{\mathrm{es,max}}$	60.00	spikes $\cdot$ s^{-1}
Parameter for efferent sympathetic activity	k_{es}	0.0675	s
Parameter for efferent sympathetic activity	$f_{\mathrm{es},\infty}$	2.10	spikes $\cdot$ s^{-1}
Parameter for efferent sympathetic activity	$f_{\mathrm{es},0}$	16.11	spikes $\cdot$ s^{-1}
**Efferent vagal pathway**			
Parameter for efferent vagal activity	$f_{\mathrm{ev},\infty}$	6.30	spikes $\cdot$ s^{-1}
Parameter for efferent vagal activity	$f_{\mathrm{ev},0}$	3.20	spikes $\cdot$ s^{-1}
Central value of afferent baroreflex firing rate	$f_{\mathrm{ab},0}$	25.00	spikes $\cdot$ s^{-1}
Offset term for efferent vagal activity	θ_{v}	-0.68	spikes $\cdot$ s^{-1}
Vagal synaptic weight factor	$W_{\mathrm{c,v}}$	0.208	-
Vagal synaptic weight factor	$W_{\mathrm{p,v}}$	-0.103	-
Parameter for efferent vagal activity	k_{ev}	7.06	spikes $\cdot$ s^{-1}
**Reflex Effectors**			
Basal splanchnic peripheral resistance	R_{sp0}	1.87	mmHg $\cdot$ s $\cdot$ mL^{-1}
Splanchnic peripheral resistance gain	$G_{\mathrm{R_{sp}}}$	0.70	mmHg $\cdot$ s $\cdot$ mL^{-1} $\cdot$ ν^{-1}
Splanchnic peripheral resistance time constant	$\tau_{\mathrm{R_{sp}}}$	6.00	s
Splanchnic peripheral resistance delay	$D_{\mathrm{R_{sp}}}$	2.00	s
Basal extra-splanchnic peripheral resistance	R_{ep0}	1.24	mmHg $\cdot$ s $\cdot$ mL^{-1}
Extra-splanchnic peripheral resistance gain	$G_{\mathrm{R_{ep}}}$	1.94	mmHg $\cdot$ s $\cdot$ mL^{-1} $\cdot$ ν^{-1}
Extra-splanchnic peripheral resistance time constant	$\tau_{\mathrm{R_{ep}}}$	6.00	s
Extra-splanchnic peripheral resistance delay	$D_{\mathrm{R_{ep}}}$	2.00	s
Skeletal muscle peripheral resistance gain	$G_{\mathrm{R_{mp}}}$	2.47	mmHg $\cdot$ s $\cdot$ mL^{-1} $\cdot$ ν^{-1}
Skeletal muscle peripheral resistance time constant	$\tau_{\mathrm{R_{mp}}}$	6.00	s
Skeletal muscle peripheral resistance delay	$D_{\mathrm{R_{mp}}}$	2.00	s
Basal splanchnic venous unstressed volume	$V_{\mathrm{u,sv0}}$	1435.40	mL
Splanchnic peripheral venous unstressed volume gain	$G_{\mathrm{V_{u,sv}}}$	-265.4	mL $\cdot$ ν^{-1}
Splanchnic peripheral venous unstressed volume time constant	$\tau_{\mathrm{V_{u,sv}}}$	20.00	s
Splanchnic peripheral venous unstressed volume delay	$D_{\mathrm{V_{u,sv}}}$	5.00	s
Basal extra-splanchnic venous unstressed volume	$V_{\mathrm{u,ev0}}$	640.73	mL
Extra-splanchnic peripheral venous unstressed volume gain	$G_{\mathrm{V_{u,ev}}}$	-74.21	mL $\cdot$ ν^{-1}
Extra-splanchnic peripheral venous unstressed volume time constant	$\tau_{\mathrm{V_{u,ev}}}$	20.00	s
Extra-splanchnic peripheral venous unstressed volume delay	$D_{\mathrm{V_{u,ev}}}$	5.00	s
Basal skeletal muscle venous unstressed volume	$V_{\mathrm{u,mv0}}$	503.26	mL
Skeletal muscle peripheral venous unstressed volume gain	$G_{\mathrm{V_{u,mv}}}$	-58.29	mL $\cdot$ ν^{-1}
Skeletal muscle peripheral venous unstressed volume time constant	$\tau_{\mathrm{V_{u,mv}}}$	20.00	s
Skeletal muscle peripheral venous unstressed volume delay	$D_{\mathrm{V_{u,mv}}}$	5.00	s
Basal left ventricle maximum elastance	$E_{\mathrm{max},LV0}$	2.39	mmHg $\cdot$ mL^{-1}
Left ventricle maximum elastance gain	$G_{\mathrm{E_{max,LV}}}$	0.475	mmHg $\cdot$ mL^{-1} $\cdot$ ν^{-1}
Left ventricle maximum elastance time constant	$\tau_{\mathrm{E_{max,LV}}}$	8.00	s
Left ventricle maximum elastance delay	$D_{\mathrm{E_{max,LV}}}$	2.00	s
Basal right ventricle maximum elastance	$E_{\mathrm{max},RV0}$	1.41	mmHg $\cdot$ mL^{-1}
Right ventricle maximum elastance gain	$G_{\mathrm{E_{max,RV}}}$	0.282	mmHg $\cdot$ mL^{-1} $\cdot$ ν^{-1}
Right ventricle maximum elastance time constant	$\tau_{\mathrm{E_{max,RV}}}$	8.00	s
Right ventricle maximum elastance delay	$D_{\mathrm{E_{max,RV}}}$	2.00	s
Sympathetic heart period gain	$G_{\mathrm{T_s}}$	-0.13	s $\cdot$ ν^{-1}
Sympathetic heart period time constant	$\tau_{\mathrm{T_s}}$	2.00	s
Sympathetic heart period delay	$D_{\mathrm{T_s}}$	2.00	s
Vagal heart period gain	$G_{\mathrm{T_v}}$	0.09	s $\cdot$ ν^{-1}
Vagal heart period time constant	$\tau_{\mathrm{T_v}}$	1.50	s
Vagal heart period delay	$D_{\mathrm{T_v}}$	0.20	s
Basal heart period	T_0	0.50	s

Note: ν = spikes/s.

Table 8: **Baseline parameters in equations (158)-(165).**

Parameter	Notation	Value	Unit
Basal minimum respiratory muscle pressure	$P_{\mathrm{mus,min}\,0}$	-5.00	$\mathrm{cmH_2O}$
Basal respiration rate	RR_0	12.00	$\mathrm{breaths} \cdot \mathrm{min}^{-1}$
**Peripheral Chemoreceptors**			
Blood transport delay	D_{p}	7.00	s
Gain of P_{mus}	$G_{\mathrm{p,A}}$	1,200.00	$\mathrm{cmH_2O} \cdot \nu^{-1}$
Gain of RR	$G_{\mathrm{p,f}}$	0.8913	$\mathrm{breaths} \cdot \mathrm{min}^{-1} \cdot \nu^{-1}$
Time constant of P_{mus}	$\tau_{\mathrm{p,A}}$	$\begin{cases} 83.00 & \text{if } u_{\mathrm{p}}(t) \geq 0 \\ 10.00 & \text{otherwise} \end{cases}$	s
Time constant of RR	$\tau_{\mathrm{p,f}}$	$\begin{cases} 174.78 & \text{if } u_{\mathrm{p}}(t) \geq 0 \\ 17.526 & \text{otherwise} \end{cases}$	s
Peripheral chemoreceptor baseline firing rate	$f_{\mathrm{apc,0}}$	3.70	$\mathrm{spikes} \cdot \mathrm{s}^{-1}$
**Central Chemoreceptors**			
Blood transport delay	D_{c}	8.00	s
Gain of P_{mus}	$G_{\mathrm{c,A}}$	$\begin{cases} 850.00 & \text{if } u_{\mathrm{c}}(t) > 5 \\ 0.00 & \text{otherwise} \end{cases}$	$\mathrm{cmH_2O} \cdot \mathrm{mmHg}^{-1}$
Gain of RR	$G_{\mathrm{c,f}}$	$\begin{cases} 0.90 & \text{if } u_{\mathrm{c}}(t) > 5 \\ 0.00 & \text{otherwise} \end{cases}$	$\mathrm{breaths} \cdot \mathrm{min}^{-1} \cdot \mathrm{mmHg}^{-1}$
Time constant of P_{mus}	$\tau_{\mathrm{c,A}}$	$\begin{cases} 105.00 & \text{if } u_{\mathrm{c}}(t) > 5 \\ 30.00 & \text{otherwise} \end{cases}$	s
Time constant of RR	$\tau_{\mathrm{c,f}}$	$\begin{cases} 400.00 & \text{if } u_{\mathrm{c}}(t) > 5 \\ 35.00 & \text{otherwise} \end{cases}$	s

Note: ν = spikes/s.

Model predicted indexes in the baseline case

See **S2 Appendix - Definition of cardiovascular and cardio-respiratory indexes** for a precise definition of model output indexes.

Table 9: **Model predicted indexes and reference values.**

Index	Model	Reference	Units
**Hemodynamic variables**			
HR	67.62	69 (12) [12]	beats$\cdot$ min^{-1}
SV	97.90	112 (19) [7]	mL
$V_{LV,max}$	157.60	160 (29) [7]	mL
EF	62	63 (5) [4]	%
C_{sa}I	1.13	1.08 (0.27) [1]	mL $\cdot$ mmHg^{-1} $\cdot$ m^{-2}
E_{LV}I	4.12	4.50 (-) [15]	mmHg $\cdot$ m^2 $\cdot$ mL^{-1}
EaI	2.38	2.20 (-) [15]	mmHg $\cdot$ m^2 $\cdot$ mL^{-1}
EaI/E_{LV}I	0.58	0.58(-) [15]	-
**Pressures**			
MAP	93.03	90 (8) [12]	mmHg
CDBP	77.94	75 (8) [12]	mmHg
CSBP	109.64	108 (12) [12]	mmHg
PDBP	76.70	74 (8) [12]	mmHg
PSBP	121.83	120 (11) [12]	mmHg
MPAP	12.43	14 (3) [8]	mmHg
CVP	2.89	(0-5) [9]	mmHg
CPP	31.91	33 (10) [12]	mmHg
PPP	45.13	46 (10) [12]	mmHg
**Cardiac cycle average flow**			
CO index	3.45	2.9 (0.8) [6]	mL $\cdot$ s^{-1} $\cdot$ m^{-2}
Cerebral blood flow	13.42	12.18 (2.12) [5]	mL $\cdot$ s^{-1}
Coronary blood flow	4.53	4.5 (1.36) [16]	mL $\cdot$ s^{-1}
**Cardio-respiratory variables**			
P_{a,O_2}	85.51	75–100 [13]	mmHg
P_{a,CO_2}	40.26	35–45 [13]	mmHg
C_{v,O_2}	0.16	0.12–0.15 [13]	$mL_{gas} \cdot mL_{blood}{}^{-1}$
C_{v,CO_2}	0.51	0.52 [3]	$mL_{gas} \cdot mL_{blood}{}^{-1}$
P_{A,CO_2}	40.24	38-42 [2]	mmHg

Main model-predicted cardiac and haemodynamic indexes and reference values, mean (SD), reported in the literature. Note that peripheral pressures are derived from central pressures using a conversion factor. Notation: SV, left ventricle stroke volume; EF, left ventricle ejection fraction; C_{sa}I, total arterial compliance index; E_{LV}I, left ventricle elastance index; EaI, arterial elastance index; MAP/CSBP/CDBP, mean/systolic/diastolic central blood pressure; PSBP/PDBP, systolic/diastolic peripheral blood pressure; MPAP, mean pulmonary arterial pressure; CVP, central venous pressure; CPP, central pulse pressure; PPP peripheral pulse pressure; P_{a,O_2}, arterial blood O_2 partial pressure; P_{a,CO_2}, arterial blood CO_2 partial pressure; C_{v,O_2}, mixed venous blood O_2 concentration; C_{v,CO_2}, mixed venous blood CO_2 concentration; P_{A,CO_2}, alveolar CO_2 partial pressure.

References

[1] Abdelhammed I. Abdelhammed, Ronald D. Smith, Pavel Levy, Gerard J. Smits, and Carlos M. Ferrario. Noninvasive hemodynamic profiles in hypertensive subjects. *American Journal of Hypertension*, 18(2 Pt 2):51S–59S, February 2005.

[2] Antonio Albanese, Limei Cheng, Mauro Ursino, and Nicolas W. Chbat. An integrated mathematical model of the human cardiopulmonary system: model development. *American Journal of Physiology. Heart and Circulatory Physiology*, 310(7):H899–921, April 2016.

[3] G. J. Arthurs and M. Sudhakar. Carbon dioxide transport. *Continuing Education in Anaesthesia, Critical Care and Pain*, 5(6):207–210, December 2005.

[4] Barry A. Borlaug, Carolyn S. P. Lam, Véronique L. Roger, Richard J. Rodeheffer, and Margaret M. Redfield. Contractility and ventricular systolic stiffening in hypertensive heart disease insights into the pathogenesis of heart failure with preserved ejection fraction. *Journal of the American College of Cardiology*, 54(5):410–418, July 2009.

[5] Matthew D Ford, Noam Alperin, Sung Hoon Lee, David W Holdsworth, and David A Steinman. Characterization of volumetric flow rate waveforms in the normal internal carotid and vertebral arteries. *Physiological Measurement*, 26(4):477–488, August 2005.

[6] A. Ganau, R. B. Devereux, M. J. Roman, G. de Simone, T. G. Pickering, P. S. Saba, P. Vargiu, I. Simongini, and J. H. Laragh. Patterns of left ventricular hypertrophy and geometric remodeling in essential hypertension. *Journal of the American College of Cardiology*, 19(7):1550–1558, June 1992.

[7] Lucy E. Hudsmith, Steffen E. Petersen, Jane M. Francis, Matthew D. Robson, and Stefan Neubauer. Normal human left and right ventricular and left atrial dimensions using steady state free precession magnetic resonance imaging. *Journal of Cardiovascular Magnetic Resonance: Official Journal of the Society for Cardiovascular Magnetic Resonance*, 7(5):775–782, 2005.

[8] Edmund M. T. Lau, Laurent Godinas, Olivier Sitbon, David Montani, Laurent Savale, Xavier Jaïs, Frederic Lador, Sven Gunther, David S. Celermajer, Gérald Simonneau, Marc Humbert, Denis Chemla, and Philippe Herve. Resting pulmonary artery pressure of 21-24 mmHg predicts abnormal exercise haemodynamics. *The European Respiratory Journal*, 47(5):1436–1444, 2016.

[9] J. R. Levick. *An introduction to cardiovascular physiology / J. Rodney Levick.* Hodder Arnold, London, 5th ed. edition, 2010.

[10] F. Y. Liang, S. Takagi, R. Himeno, and H. Liu. Biomechanical characterization of ventricular–arterial coupling during aging: A multi-scale model study. *Journal of Biomechanics*, 42(6):692–704, April 2009.

[11] E. Magosso and M. Ursino. A mathematical model of CO2 effect on cardiovascular regulation. *American Journal of Physiology. Heart and Circulatory Physiology*, 281(5):H2036–2052, November 2001.

[12] Carmel M. McEniery, Yasmin, Barry McDonnell, Margaret Munnery, Sharon M. Wallace, Chloe V. Rowe, John R. Cockcroft, and Ian B. Wilkinson. Central Pressure: Variability and Impact of Cardiovascular Risk Factors: The Anglo-Cardiff Collaborative Trial II. *Hypertension*, 51(6):1476–1482, June 2008.

[13] William T McGee, William R McIvor, Simon J Davies, Tami Franco-McKinney, and John A Frazier. Quick Guide to Cardiopulmonary Care. *Edwards*, 2023.

[14] J. P. Mynard, M. R. Davidson, D. J. Penny, and J. J. Smolich. A simple, versatile valve model for use in lumped parameter and one-dimensional cardiovascular models. *International Journal for Numerical Methods in Biomedical Engineering*, 28(6-7):626–641, 2012.

[15] Samer S. Najjar, Steven P. Schulman, Gary Gerstenblith, Jerome L. Fleg, David A. Kass, Frances O'Connor, Lewis C. Becker, and Edward G. Lakatta. Age and gender affect ventricular-vascular coupling during aerobic exercise. *Journal of the American College of Cardiology*, 44(3):611–617, August 2004.

[16] Shingo Sakamoto, Saeko Takahashi, Ahmet U. Coskun, Michail I. Papafaklis, Akihiko Takahashi, Shigeru Saito, Peter H. Stone, and Charles L. Feldman. Relation of Distribution of Coronary Blood Flow Volume to Coronary Artery Dominance. *The American Journal of Cardiology*, 111(10):1420–1424, May 2013.

[17] M. Ursino. Interaction between carotid baroregulation and the pulsating heart: a mathematical model. *The American Journal of Physiology*, 275(5):H1733–1747, November 1998.

[18] M. Ursino and E. Magosso. Acute cardiovascular response to isocapnic hypoxia. I. A mathematical model. *American Journal of Physiology. Heart and Circulatory Physiology*, 279(1):H149–165, July 2000.

[19] Mauro Ursino and Elisa Magosso. A theoretical analysis of the carotid body chemoreceptor response to O2 and CO2 pressure changes. *Respiratory Physiology & Neurobiology*, 130(1):99–110, March 2002.
