# Supplementary material for: Predicting in-hospital indicators from wearable-derived signals for cardiovascular and respiratory disease monitoring: An in silico study
Source: PLOS Digit Health. 2025 Oct 14;4(10):e0001041. doi: 10.1371/journal.pdig.0001041 (PMC12520384; doi:10.1371/journal.pdig.0001041)
Supplement: S2 Appendix — A precise description of the computation of the extracted model output variables. (PDF) [file pdig.0001041.s002.pdf]

Supplementary Material for: Predicting in-hospital indicators from wearable-derived signals for cardiovascular and respiratory disease monitoring: an in silico study, by Bianca Maria Laudenzi, Alberto Cucino, Sergio Lassola, Eleonora Balzani, Lucas Omar Müller. Corresponding author: Bianca Maria Laudenzi, biancamaria.laudenzi@unitn.it

Definition of cardiovascular and cardio-respiratory indexes

All indexes are computed over the interval $[T_{\text{ini}}, T_{\text{end}}]$, with $I = T_{\text{end}} - T_{\text{ini}}$, $I \in \mathbb{N}$. The fraction of the cardiac cycle is represented by a dimensionless variable $u(t)$, which ranges from 0 to 1. Each heart period (HP_i) within $[T_{\text{ini}}, T_{\text{end}}]$ is identified as the interval between two consecutive minimums of $u(t)$, i.e. $[t_i, t_{i+1}]$. Thus, $\text{HP}_i = t_{i+1} - t_i$.

Heart Rate (HR). The HR is computed as

$$\text{HR} = \frac{\text{number of HP}_\text{i}}{I} \cdot 60$$

Central arterial blood pressures: systolic, diastolic, pulse pressure, mean (CSBP, CDBP, CPP, MAP). These indexes are calculated from the systemic arterial pressure $P_{\text{sa}}(t)$ curve as

$$\text{CSBP} = \frac{1}{I} \sum_{i=1}^{I} \max_{t \in [t_i,\, t_{i+1}]} P_{\text{sa}}(t)$$

$$\text{CDBP} = \frac{1}{I} \sum_{i=1}^{I} \min_{t \in [t_i,\, t_{i+1}]} P_{\text{sa}}(t)$$

$$\text{CPP} = \frac{1}{I} \sum_{i=1}^{I} \left[\max_{t \in [t_i, t_{i+1}]} P_{\text{sa}}(t) - \min_{t \in [t_i, t_{i+1}]} P_{\text{sa}}(t) \right]$$

$$\text{MAP} = \frac{1}{I} \sum_{i=1}^{I} \left[\frac{1}{\text{HP}_i} \int_{t_i}^{t_{i+1}} P_{\text{sa}}(t)\, dt \right]$$

Central venous pressure (CVP). The CVP is calculated from the cardiac cycle-averaged thoracic vein pressure P_{tv} as

$$\text{CVP} = \frac{1}{I} \sum_{i=1}^{I} \left[\frac{1}{\text{HP}_i} \int_{t_i}^{t_{i+1}} P_{\text{tv}}(t)\, dt \right]$$

Left ventricle stroke volume (SV). The SV is computed from the cardiac cycle-averaged flow through the aortic valve q_{AV} as

$$\text{SV} = \frac{1}{I} \sum_{i=1}^{I} \left[\int_{t_i}^{t_{i+1}} q_{\text{AV}}(t)\, dt \right]$$

Left ventricle cardiac output (CO). The CO is computed from the cardiac cycle-averaged flow through the aortic valve q_{AV} as

$$\text{CO} = \frac{1}{I} \sum_{i=1}^{I} \left[\frac{1}{\text{HP}_i} \int_{t_i}^{t_{i+1}} q_{\text{AV}}(t)\, dt \right]$$

Left ventricle ejection fraction (EF). The EF is computed from the left ventricle volume curve V_{LV} as

$$\text{EF} = \frac{1}{I} \sum_{i=1}^{I} \left[\frac{\text{LVEDV}_i - \text{LVESV}_i}{\text{LVEDV}_i} \right]$$

with LVEDV_i and LVESV_i being the left ventricle end-diastolic and end-systolic volumes for each HP_i respectively, and are defined as

$$\mathrm{LVEDV}_i = \max_{t\in[t_i,\, t_{i+1}]} V_{\mathrm{LV}}(t) \qquad \text{and}$$
$$\mathrm{LVESV}_i = \min_{t\in[t_i,\, t_{i+1}]} V_{\mathrm{LV}}(t)$$

Arterial oxygen saturation ($\mathbf{S_{a,O_2}}$). The $\mathrm{S}_{\mathrm{a,O_2}}$ is computed from the arterial oxygen saturation curve $S_{\mathrm{a,O_2}}$ as

$$\mathrm{S}_{\mathrm{a,O_2}} = \frac{1}{I}\sum_{i=1}^{I}\left[\frac{1}{\mathrm{HP}_i}\int_{t_i}^{t_{i+1}} S_{\mathrm{a,O_2}}(t)\,dt\right]$$

Arterial partial pressure of oxygen ($\mathbf{P_{a,O_2}}$). The $\mathrm{P}_{\mathrm{a,O_2}}$ is computed from the arterial partial pressure curve of $\mathrm{O_2}$ $P_{\mathrm{a,O_2}}$ as

$$\mathrm{P}_{\mathrm{a,O_2}} = \frac{1}{I}\sum_{i=1}^{I}\left[\frac{1}{\mathrm{HP}_i}\int_{t_i}^{t_{i+1}} P_{\mathrm{a,O_2}}(t)\,dt\right]$$

Arterial partial pressure of carbon dioxide ($\mathbf{P_{a,CO_2}}$). The $\mathrm{P}_{\mathrm{a,CO_2}}$ is computed from the arterial partial pressure curve of $\mathrm{CO_2}$ $P_{\mathrm{a,CO_2}}$ as

$$\mathrm{P}_{\mathrm{a,CO_2}} = \frac{1}{I}\sum_{i=1}^{I}\left[\frac{1}{\mathrm{HP}_i}\int_{t_i}^{t_{i+1}} P_{\mathrm{a,CO_2}}(t)\,dt\right]$$
