# Supplementary material for: Predicting in-hospital indicators from wearable-derived signals for cardiovascular and respiratory disease monitoring: An in silico study
Source: PLOS Digit Health. 2025 Oct 14;4(10):e0001041. doi: 10.1371/journal.pdig.0001041 (PMC12520384; doi:10.1371/journal.pdig.0001041)
Supplement: S3 Appendix — Convergence, distributions, post-processing filter and CVRD-classification. (PDF) [file pdig.0001041.s003.pdf]

Supplementary Material for: Predicting in-hospital indicators from wearable-derived signals for cardiovascular and respiratory disease monitoring: an in silico study, by Bianca Maria Laudenzi, Alberto Cucino, Sergio Lassola, Eleonora Balzani, Lucas Omar Müller. Corresponding author: Bianca Maria Laudenzi, biancamaria.laudenzi@unitn.it

Virtual population 2 results

Fig. 1 shows the heat-map of the Total Sobol indices for the selected 10 model input parameters (y-axis) over all clinically relevant output variables (x-axis) of the virtual population (VP) 2 generated with 4096 sampling points. Darker colors refer to higher interaction between inputs and outputs. The indexes for each parameter are normalized so that they sum up to 1 (representing 100% of the output variance). Indexes lower than 0.01 are not annotated in the heat-map. The variance-based global sensitivity analysis identified the total blood volume, V_{tot}, and the venous unstressed volume, $V_{\mathrm{u,ven}}$, as the most significant parameter for the bio-signals of the cardiovascular system, i.e. HR, CSBP, CDBP, CVP, SV, CO, EF. Moreover, the basal heart period, T_0, has a significant influence on HR, while the ventricles elastances parameters ($E_{\mathrm{max,LV}}$, $k_{\mathrm{E,LV}}$, $k_{\mathrm{E,RV}}$) greatly influence EF. Whereas, the inspired fraction of oxygen, $F_{\mathrm{iO_2}}$, the CO_2 dissociation constant, $k_{\mathrm{CO_2}}$, and the CO_2 saturation concentration, $C_{\mathrm{sat,CO_2}}$, are the most significant parameter for the cardio-respiratory bio-signals, i.e. $\mathrm{S_{a,O_2}}$, $\mathrm{P_{a,O_2}}$ and $\mathrm{P_{a,CO_2}}$.

Fig. 2 shows the distributions of the model output among VP 2 generated with 4096 sampling points. The minimum, the mean, and the maximum of each variable of the virtual database are shown in the x-axes. The translucent blue bands over the histograms indicate that the variable does not meet the filter criteria, and therefore the simulation is discarded from the VP. The other translucent colored bands (red, orange, green, yellow, and purple) refer to the different cardio-vascular and -respiratory diseases (CVRD). The shapes of the distributions vary depending on the physiological variable, with some showing clear peaks (e.g., HR, $\mathrm{P_{a,CO_2}}$), while others exhibit more uniform or flat patterns (e.g., CPP, SV, CO). All variables are well distributed within their respective reference ranges, with the exception of $\mathrm{S_{a,O_2}}$, where a broader range would have been preferable. Moreover, not all filter criteria apply across the VP. For example, the filter criteria do not apply to $\mathrm{S_{a,O_2}}$. Similarly, across all simulations, the upper cutoff values for CPP and EF are never reached. In particular, Fig. 3 shows, on the left, the simulations that have failed the post-processing filter criteria among the 4096 simulations and, on the right, the percentage of failed simulations, for each output variable, among the simulations that have failed the filter criteria. Out of 4096 simulations, 2760 failed the filter criteria, therefore the final VP 2 is composed of 1336 subjects. Additionally, we note that the majority of failed simulations are due to too high $\mathrm{P_{a,O_2}}$, too low MAP, CSBP and CDBP, too low CVP, or $\mathrm{P_{a,CO_2}}$. Fig. 4 shows the pie-chart of the CVRD classification among the simulations that have not failed the filter criteria, i.e. the final VP 2. Among those, we can distinguish between healthy subjects (31.5 %), hypertensive patients (23.3 %), hyper- or hypo-ventilating ones (14.0 % and 7.3 % respectively), patients with reduced cardiac function (0.1 %), or combinations of those. Overall, the final distribution ranges of the variables of VP 2 ensure a good level of heterogeneity, contributing to a diverse and realistic representation of the data. This allows us to simulate a variety of scenarios, including a sufficient spectrum pathological conditions.

Notation HR: heart rate; CSBP: central systolic arterial blood pressure; CDBP: central diastolic arterial blood pressure; CPP: central pulse pressure; MAP: central mean arterial pressure; CVP: central venous pressure; SV: left ventricle stroke volume; CO: left ventricle cardiac output; EF: left ventricle ejection fraction; $\mathrm{S_{a,O_2}}$: arterial oxygen saturation; $\mathrm{P_{a,O_2}}$: arterial partial pressure of oxygen; $\mathrm{P_{a,CO_2}}$: arterial partial pressure of carbon dioxide.

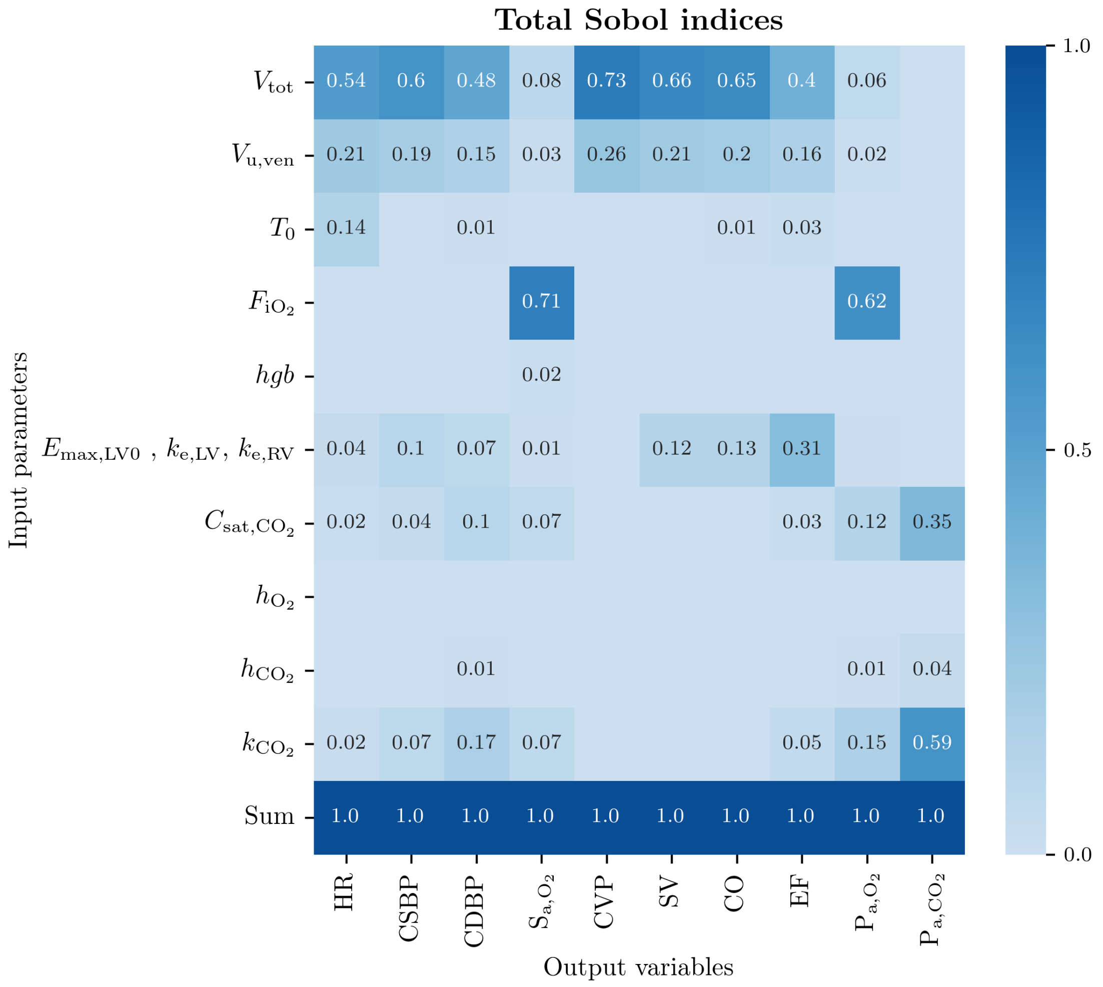

Figure 1: **Global sensitivity analysis.** Total effects of input parameters (y-axis) on model outputs (x-axis), normalized between 0 and 1, for VP 2. Parameters abbreviations: V_{tot}: total blood volume; $V_{\mathrm{u,ven}}$ venous unstressed volume; T_0: basal cardiac cycle; $F_{\mathrm{iO_2}}$: inspired fraction of O_2; hgb: blood hemoglobin content; $E_{\mathrm{max,LV}}$, $k_{\mathrm{E,LV}}$, $k_{\mathrm{E,RV}}$: left and right ventricle parameters for elastances; $C_{\mathrm{sat,\ CO_2}}$: maximum concentration of hemoglobin-bound CO_2; $h_{\mathrm{O_2}}$, $h_{\mathrm{CO_2}}$, $k_{\mathrm{CO_2}}$: empirical parameters for O_2/CO_2 dissociation curve.

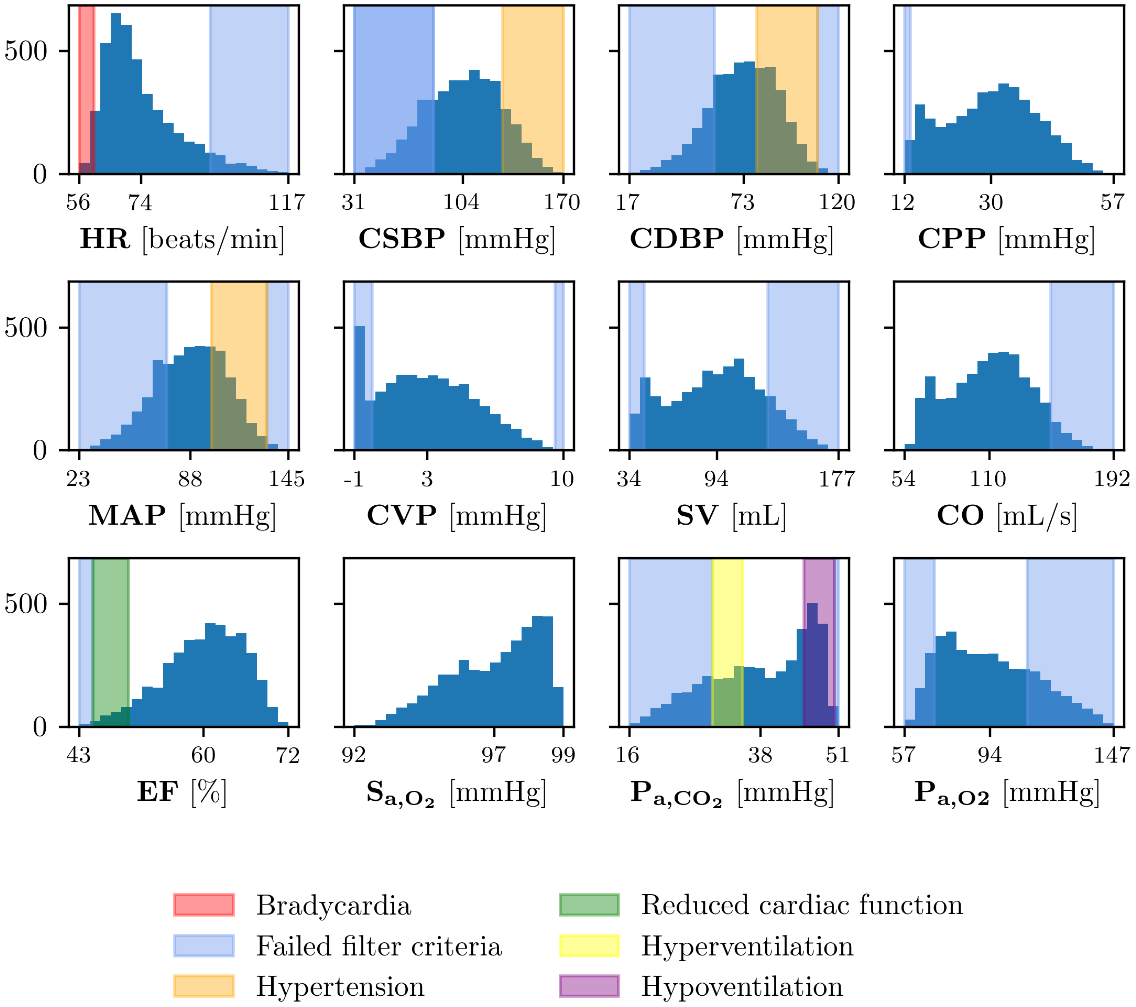

Figure 2: **Virtual population distribution.** Frequency (y-axes) of variables of interest (x-axes) across VP 2. Translucent blue bands indicate that the variable does not meet the filter criteria; translucent colored bands (red, orange, green, yellow, and purple) refer to the different CVRD.

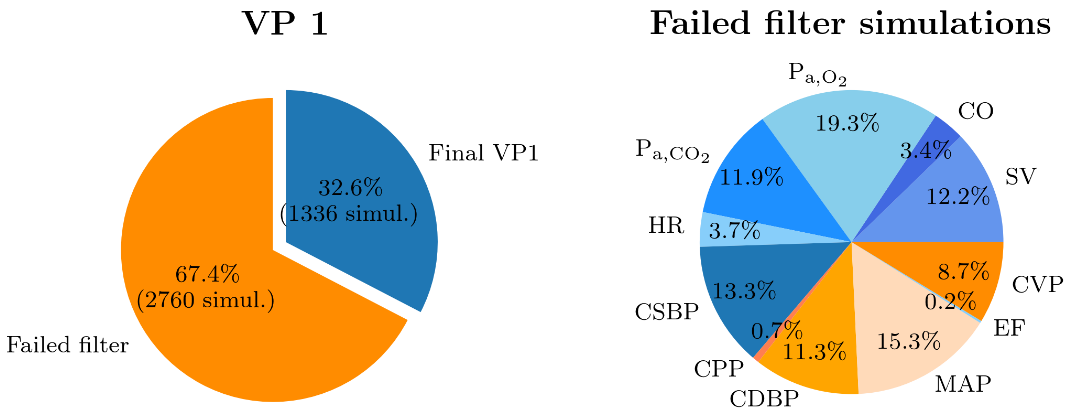

Figure 3: **Post-processing filter criteria.** On the left, the simulations that have failed the post-processing filter criteria among all 4096 simulations of VP 2. On the right, the percentage of failed simulations, for each output variable, among the simulations that have failed the filter criteria of VP 2.

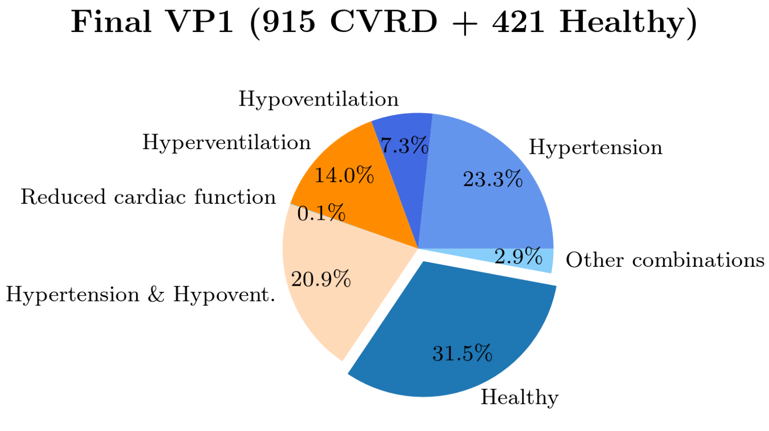

Figure 4: **CVRD classification.** CVRD classifications among the simulations that have not failed the filter criteria of VP 2.
